# Supplementary material for: Circular RNA TTN Acts As a miR-432 Sponge to Facilitate Proliferation and Differentiation of Myoblasts via the IGF2/PI3K/AKT Signaling Pathway
Source: Mol Ther Nucleic Acids. 2019 Oct 25;18:966–80. doi: 10.1016/j.omtn.2019.10.019 (PMC6881651; doi:10.1016/j.omtn.2019.10.019)
Supplement: Document S1. Tables S1 and S2, Text S1, and Figures S1–S7 [file mmc1.pdf]

OMTN, Volume *18*

Supplemental Information

Circular RNA TTN Acts As a miR-432 Sponge to Facilitate Proliferation and Differentiation of Myoblasts via the IGF2/PI3K/AKT Signaling Pathway

Xiaogang Wang, Xiukai Cao, Dong Dong, Xuemei Shen, Jie Cheng, Rui Jiang, Zhaoxin Yang, Shujun Peng, Yongzhen Huang, Xianyong Lan, Ibrahim Elsaeid Elnour, Chuzhao Lei, and Hong Chen

Supplemental Information

Table S1. Primers for qPCR

Name	Forward primer 5'→3'	Reverse primer 5'→3'
CircTTN	AAAGAACTTCCACCTCCTAAA	CGACAACCTTTTTAGCATCTT
Bta-miR-432	CGGCTCTTGGAGTAGGTCATT	GCAGGGTCCGAGGTATTC
miR-432-RT	GTCGTATCCAGTGCAGGGTCCGAGGTATTCGCACTGGATACGACCCACCC	
U6	GCTTCGGCAGCACATATACTAAAAT	CGCTTCACGAATTTGCGTGTCAT
GAPDH	TGAGGACCAGGTTGTCTCCTGCG	CACCACCCTGTTGCTGTAGCCA
MYF5	CTGCAGGAGCTGCTTAGGGA	AGGCATGCCATCAGAGCAAC
MYOD	CGCTCCAACTGTTCCGACG	AAAGGGCATGTGGGAAGGAG
MYOG	AGGGGATCATCTGCTCCCAG	ATCCCGGCAGACAATCTCAG
MYHC	GGAGGCTGATGAACAAGCCAA	GAAGTCTCGGGTCTTTGCTCT
PCNA	TCCAGAACAAGAGTATAGC	TACAACAGCATCTCCAAT
CDK2	TCTTTGCTGAGATGGTGACCC	CATCTTCATCCAGGGGAGGC
CyclinD1	CCGTCCATGCGGAAGATC	CAGGAAGCGGTCCAGGTAG
TTN	AGGGCAATTACAGATTGGTGTG	AAACTGGCAGGTCATGGTACA
IGF2	CCGCAGGAGCTGAGACATCAA	CGGTCCCCACAGACAAACTG
IRS1	TTCGCCTACCATTTCCCACC	TGGGTAAGCGGGCATCATTT
PI3K	AGCGCTGAGCAGTGTATCTT	AACCACGGGGCTCTTGAAAT
PDK1	GAAAATGCTAGGCGTCTGTGT	CACCTCCTCGGTCACTCATC
AKT	TCCCCCAGTTCTCCTACTCG	TCCTCTCCATCCTGTGTTGG

Table S2. Primers for vector construction

Name	Primer sequence 5'→3'
PCD2.1-circTTN-F	GGGGTACCAAGAGACCCAGGTAGAAGCTA
PCD2.1-circTTN-R	CGGGATCCCGTAATCTTCCTCTGGTTCCT
psiCHECK2-circTTN-F	CCGCTCGAGAAGAGACCCAGGTAGAAGCTA
psiCHECK2-circTTN-R	ATAAGAATGCGGCCGCCGTAATCTTCCTCTGGTTCCT
psiCHECK2-miR-432-Sensor-F	TCGACCACCCAATGACCTACTCCAAGACCACCCAATGACCTACTCCAAGA
psiCHECK2-miR-432-Sensor-R	GGCCTCTTGGAGTAGGTCATTGGGTGGTCTTGGAGTAGGTCATTGGGTGG
psiCHECK2-IGF2-W-F	CCGCTCGAGTGCAGGTAGGCTTGTCCTTG
psiCHECK2-IGF2-W-R	ATAAGAATGCGGCCGCTTGGAGTGTGGGGGTGTTTT
IGF2-MUT-F	CTGCTCCTCCCAAGAGTTTCCATCA
IGF2-MUT-R	TGATGGAAACTCTTGGGAGGAGCAG
psiCHECK2-IGF1-W-F	GAAAGAGTCTGGCCAAAACGG
psiCHECK2-IGF1-W-R	CAGATGTTTGTTTCTTCAGCGAG
IGF1-MUT-F	GCTCCCGCATTATGCCTTTAGGGAA
IGF1-MUT-R	TTCCCTAAAGGCATAATGCGGGAGC

Text S1. Sequence of cattle circTTN

>circTTN

TGACCAAAAAAGCTGTGAAGAAAGATGCTAAAAAGGTTGTCGCAAAGCCCAAGGAAGAG

GCACCGCCACCTAAAGTTATAGAGGTTCCCAAGAAGCCACCACGTCCTACTGCCTTAATTCC

AGCAGAAGCACCTGAAATTATTGATGTATCCTCCAAGGCTGAAGAAGTGAAAATAACGACC

ATAACCAGAAAGAAAGAGGTTCAGAAAGAAAAAGAAGCTGTGTATGAGAGAAAGCAAGC

AGTCTACGAGGAGAAGAAAGTTTACATAGAATCTTTGGAAGAACCTTACGATGAGCTAGAG

GTGGAAACGTATACGGAGCCATATGAAGAACCTTATTATGAGGAACCAGAGGAAGATTACG

AAGAGACCCAGGTAGAAGCTAAGAGGGAAGTTCATGAGGAATGGGAAGAAGATTTTGAAG

AAGGGCAAGAATACTATGAAAGGGAGGAAGGTTATGACGAAGGAGAGGAAGAGTGGGAG

GAGACTTACCAAGAGAGAGAAGTCGTCCAAGTTCAAAAGGAGGTTTATGAAGAATCACGT

GAGAGGAAAATTCCAGCAAAAGTACCTGAAAAGAAAGAACTTCCACCTCCTAAAGTTGTA

AAGAAGCCTGTAGTTGAAAAAATTGAAAAGACTTCTCGAAGAATGGAGGAAGAAAAAGTT

CAAGTCACCAAAG

Figure S1

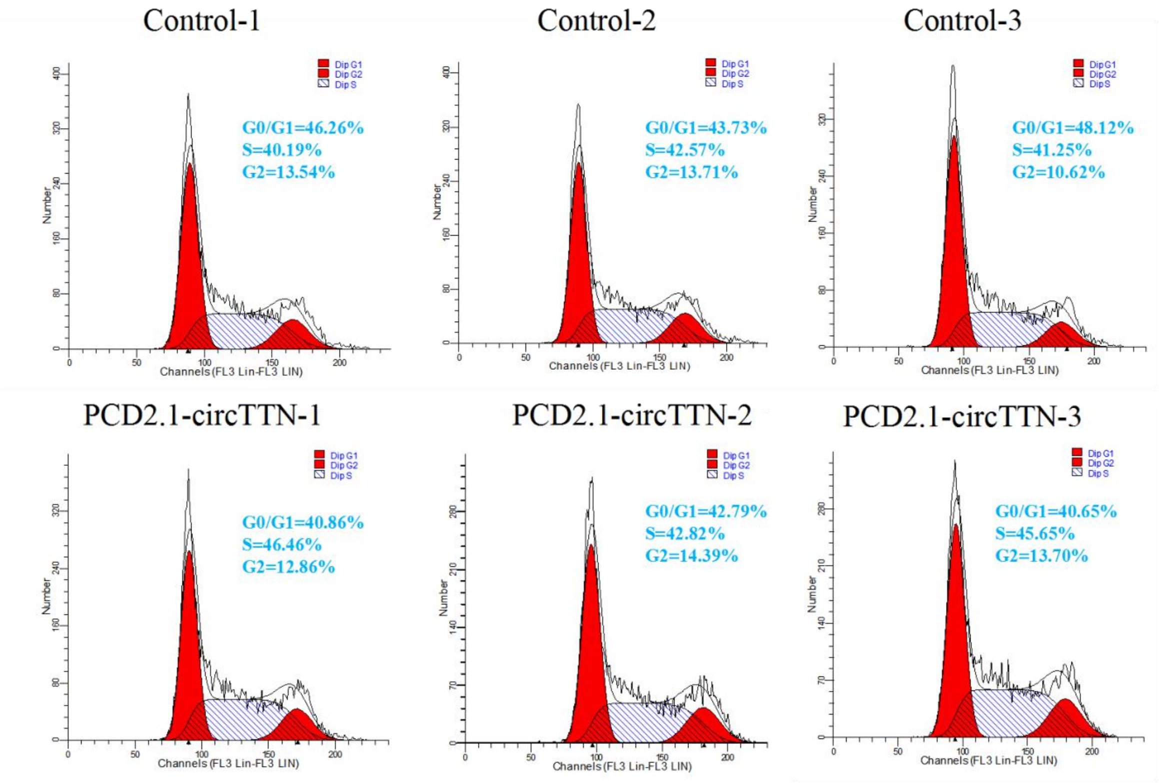

Figure S1. Bovine primary myoblasts were transfected with PCD2.1-circTTN, and cell phases were analyzed by flow cytometry

Figure S2

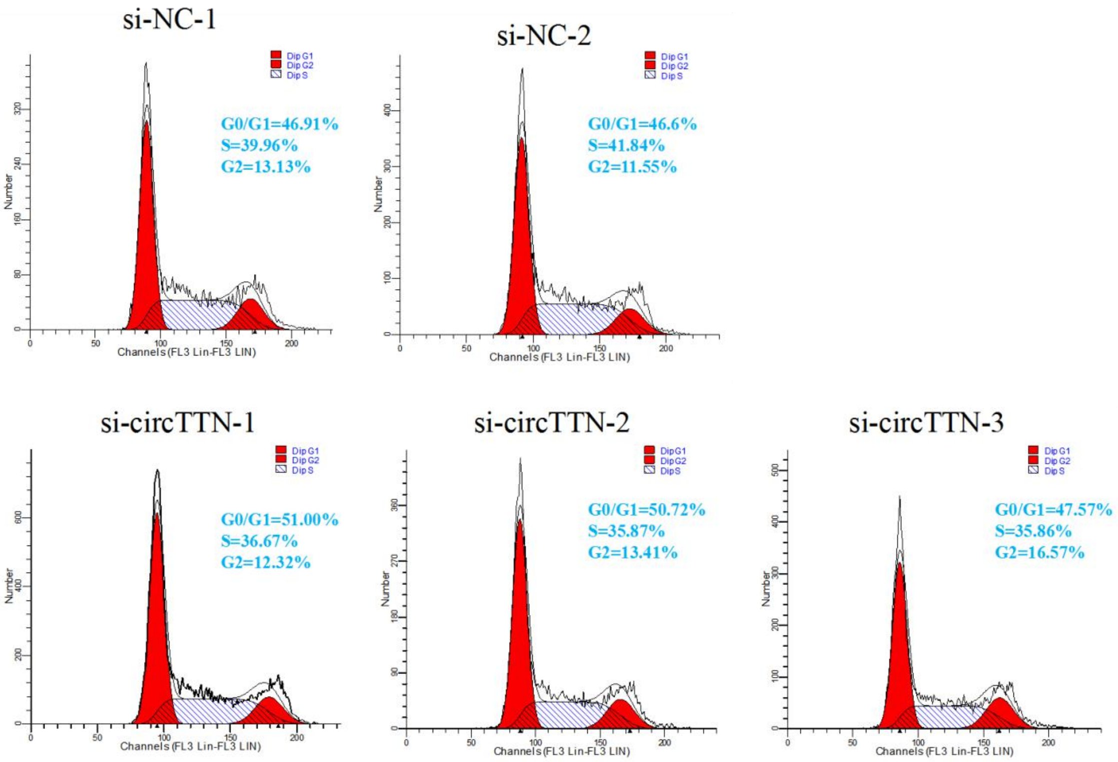

Figure S2. Bovine primary myoblasts were transfected with si-circTTN, and cell phases were analyzed by flow cytometry

Figure S3

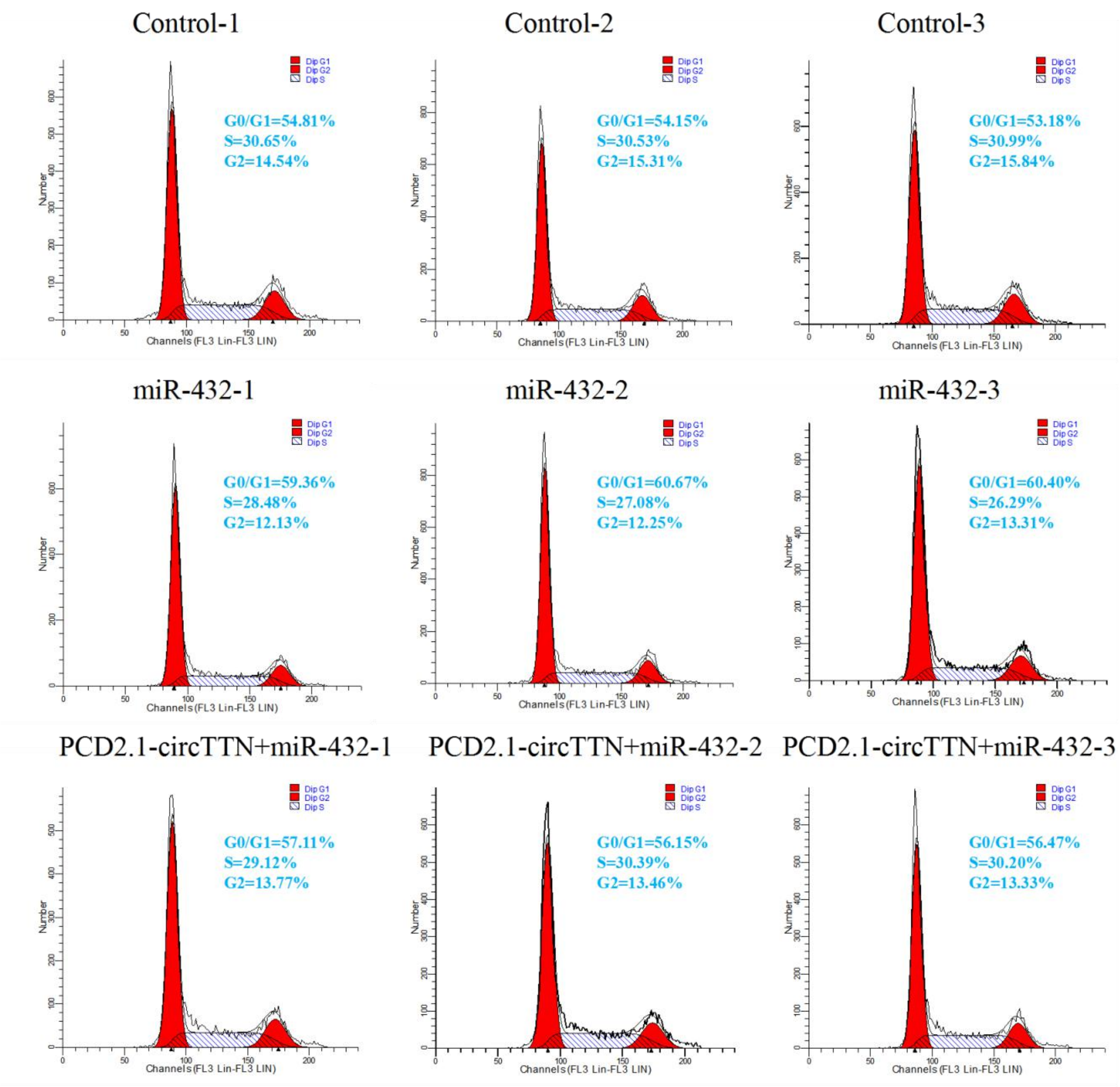

Figure S3. Bovine primary myoblasts were transfected with miR-432 mimic and/or PCD2.1-circTTN, and cell phases were analyzed by flow cytometry

Figure S4

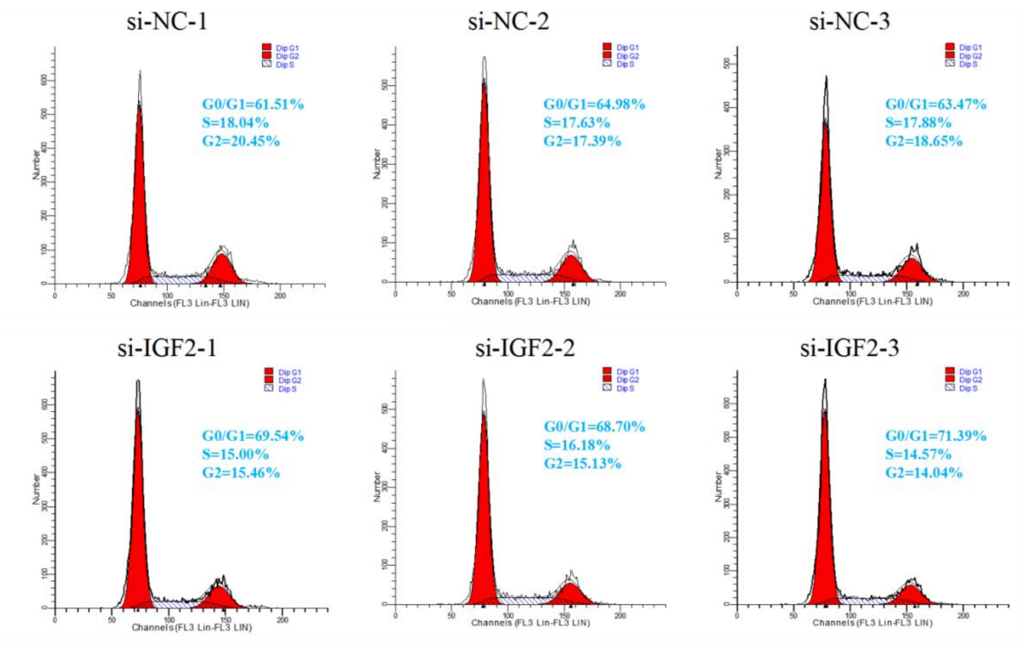

Figure S4. Bovine primary myoblasts were transfected with si-IGF2, and cell phases were analyzed by flow cytometry

Figure S5

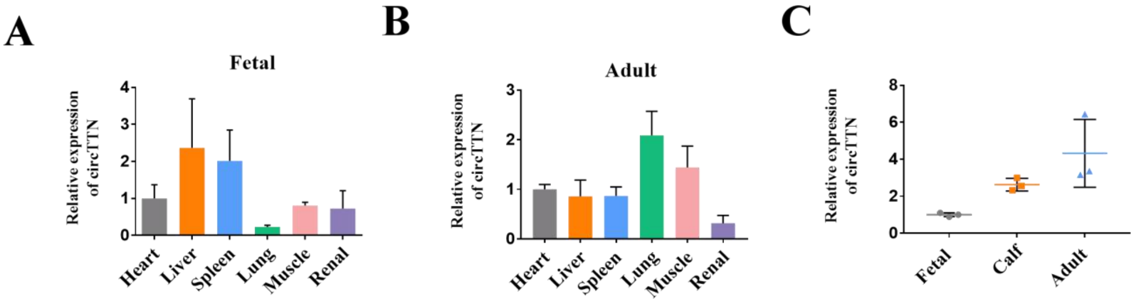

(A and B) The expression of circTTN in different tissues of Qinchuan cattle at embryonic (A) and adult (B) stage. (C) The expression of circTTN in skeletal muscle of cattle at the fetal stage, calf stage and adult stage.

Figure S6

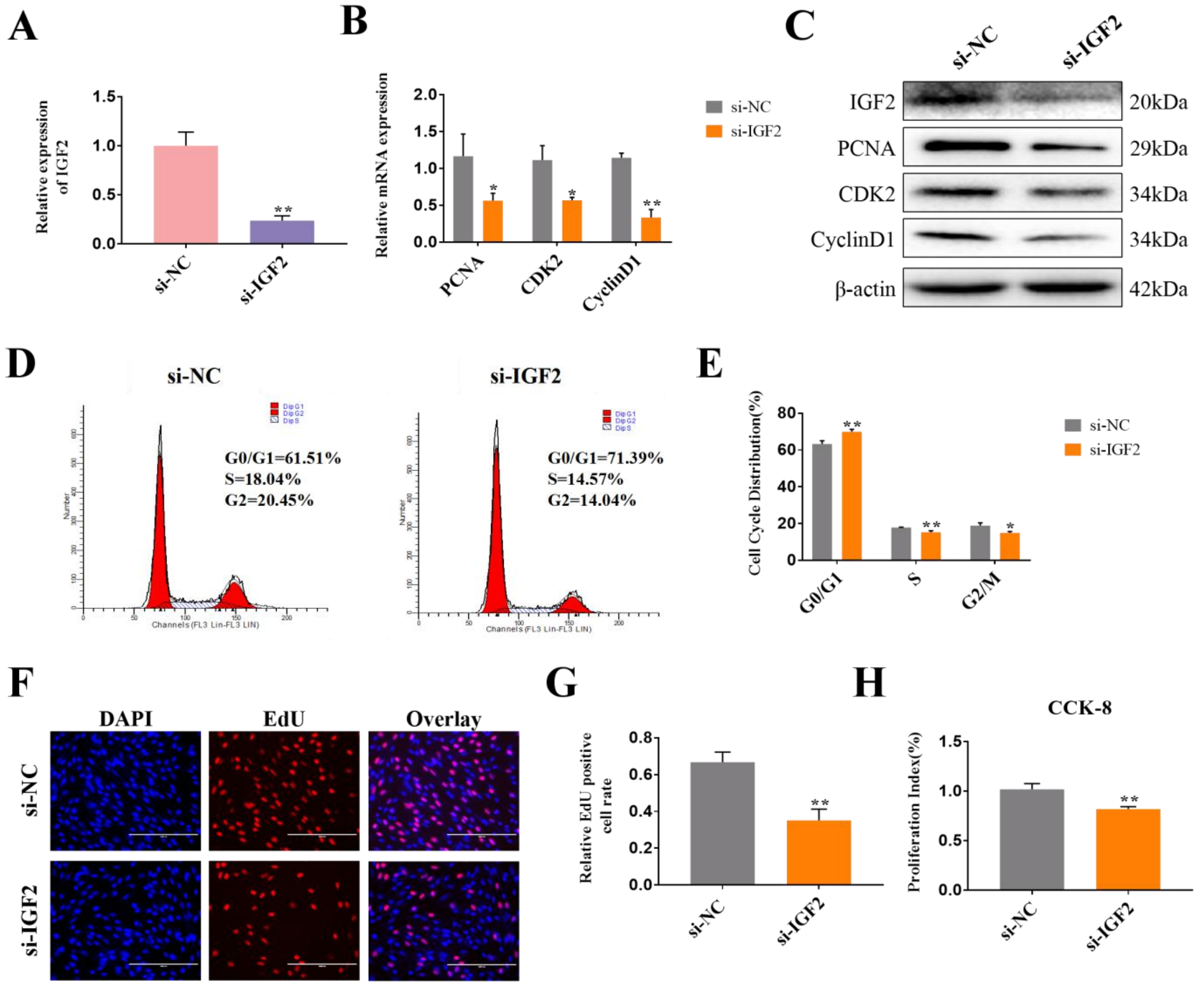

Figure S6 Effect of *IGF2* Knockdown on Proliferation of Bovine Primary Myoblasts

(A) The interference efficiency of the si-*IGF2* is detected by RT-qPCR. (B) Detection of the expression levels of the cell proliferation genes mRNA (*PCNA*, *CDK2*, *Cyclin D1*) by RT-qPCR. (C) Detection of PCNA, CDK2, and Cyclin D1 protein expression levels by western blot analysis, β-actin acts as an internal control gene. (D and E) Bovine primary myoblasts were transfected with si-*IGF2*, and cell phases were analyzed by flow cytometry (D) and counted (E). (F and G) Cell proliferation was detected by 5-ethynyl-2'-deoxyuridine (EdU) (F) and counted using Image J (G). Scale bar indicates 200 μm. (H) Cell proliferation index was detected by cell counting kit-8 (CCK-8) assay. Data are presented as means ± SEM for three individuals. *$P < 0.05$; **$P < 0.01$.

Figure S7

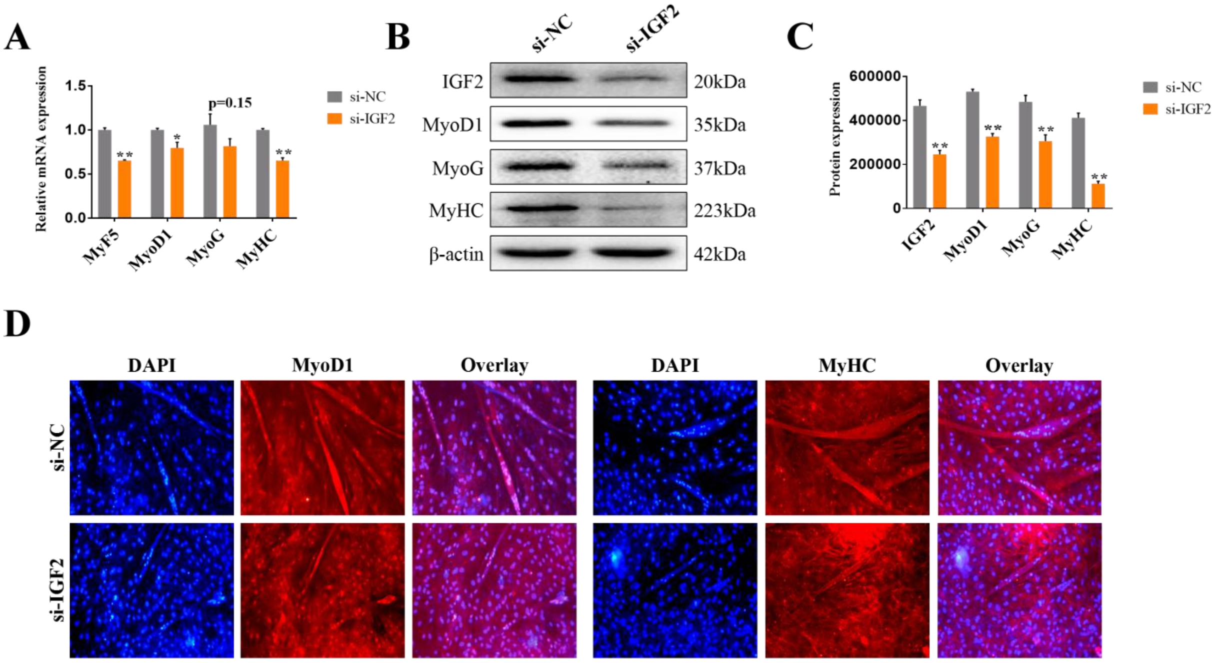

Figure S7 Effect of *IGF2* Knockdown on Differentiation of Bovine Primary Myoblasts

(A) Transfection of the si-*IGF2* and the expression levels of myogenic differentiation marker genes *MyF5*, *MyoD1*, *MyoG*, and *MyHC* mRNA were detected by RT-qPCR. (B and C) MyoD1, MyoG, and MyHC protein levels were detected by western blot analysis (B), and protein band density was also analyzed by ImageJ (C). (D) Bovine primary myoblasts were transfected with si-*IGF2* and cell differentiation was measured by immunofluorescence (MyoD1 (left) and MyHC (right)) and observed under a fluorescence microscope. Scale bar indicates 200 μm. Data are presented as means ± SEM for three individuals. *$P < 0.05$; **$P < 0.01$.
