# Supplementary material for: Circular RNA TTN Acts As a miR-432 Sponge to Facilitate Proliferation and Differentiation of Myoblasts via the IGF2/PI3K/AKT Signaling Pathway
Source: Mol Ther Nucleic Acids. 2019 Oct 25;18:966–80. doi: 10.1016/j.omtn.2019.10.019 (PMC6881651; doi:10.1016/j.omtn.2019.10.019)
Supplement: Document S2. Article plus Supplemental Information [file mmc2.pdf]

Molecular Therapy
Nucleic Acids
Original Article

Circular RNA TTN Acts As a miR-432 Sponge to Facilitate Proliferation and Differentiation of Myoblasts via the IGF2/PI3K/AKT Signaling Pathway

Xiaogang Wang,[1,2] Xiukai Cao,[1,2] Dong Dong,[1] Xuemei Shen,[1] Jie Cheng,[1] Rui Jiang,[1] Zhaoxin Yang,[1] Shujun Peng,[1] Yongzhen Huang,[1] Xianyong Lan,[1] Ibrahim Elsaeid Elnour,[1] Chuzhao Lei,[1] and Hong Chen[1]

[1]Shaanxi Key Laboratory of Animal Genetics, Breeding and Reproduction, College of Animal Science and Technology, Northwest A&F University, Shaanxi 712100, China

Circular RNAs (circRNAs) are ubiquitous endogenous RNA found in various organisms that can regulate gene expression in eukaryotes. However, little is known about potential roles for circRNAs in muscle development. We analyzed circRNA sequencing data of bovine skeletal muscle tissue and found differential expression of *circTitin* (*circTTN*) in fetal and adult bovine muscle tissue. We then further studied the role of *circTTN* in bovine muscle development. Overexpression and inhibition of *circTTN* together elicited its promoting roles in proliferation and differentiation of bovine primary myoblasts. Mechanistically, *circTTN* showed interaction with miR-432 by luciferase screening and RNA immunoprecipitation (RIP) assays. Additionally, miR-432 is a regulator of insulin-like growth factor 2 (*IGF2*), as indicated by luciferase activity, quantitative real-time PCR, and western blotting assays. Increased miR-432 expression inhibited the expression of *IGF2*, but this effect was remitted by *circTTN*. Conclusively, our results showed that *circTTN* promoted proliferation and differentiation of bovine primary myoblasts via competitively combining with miR-432 to activate the *IGF2*/phosphatidylinositol 3-kinase (*PI3K*)/*AKT* signaling pathway.

INTRODUCTION

Circular RNAs (circRNAs) are widespread and diverse endogenous RNAs with covalently closed continuous loop structures, generated by back-splicing of a single pre-mRNA, making these RNAs more stable than linear RNA.[1,2] The first circRNA was identified in human cells in the early 1990s.[3] Although circRNAs were discovered decades ago, they were originally considered byproducts of spliceosome-mediated splicing errors and thought to lack any significant function.[4] However, recent high-throughput sequencing and novel computational approaches have identified a large number of circRNAs within the transcriptome, suggesting potential roles for these RNAs in development.[5–9] These circRNAs have extremely abundant microRNA (miRNA) binding sites and thus act as a competitive endogenous RNA (ceRNA) to regulate miRNA expression.[10,11] For example, two circRNAs, *ciRS-7/CDR1as* and *Sry*, were reported to contain multiple miRNA binding sites.[6,12] Besides, it has been proposed that some circRNAs may "sponge" other factors, such as RNA binding proteins (RBPs).[13] Recently, a novel subclass of circRNAs has been described as exon-intron circRNAs (EIciRNAs). These EIciRNAs interact with RNA polymerase II and U1 small nuclear ribonucleoproteins (snRNPs) and can act in *cis* to induce host-gene transcription in the nucleus.[14]

Skeletal muscle is one of the most dynamic and plastic tissues in the human body, playing a crucial role in movement, metabolism, and homeostasis, accounting for about 40% of adult body weight.[15,16] Skeletal muscle fibers are formed by the fusion of multiple mononuclear myoblasts[17] in a process that is regulated by multiple factors during myogenesis. Myogenesis is regulated by myogenic regulatory factors (MRFs)[18,19] and various noncoding RNAs, such as miRNAs and long noncoding RNAs (lncRNAs).[20–23] Recent work has examined potential roles for circRNAs during myogenesis in a variety of organisms. For example, the mouse ortholog *circZfp609* acts as a decoy for miR-194-5p to promote expression of *BCLAF1* and thereby suppress myoblast differentiation;[24] *circFGFR4* inhibits bovine primary myoblast differentiation and apoptosis by sponging miR-107;[25] and chicken *circSVIL* promotes myoblast proliferation and differentiation by sponging miR-203 and increasing expression of targets *c-JUN* and *MEF2C*.[26] Studies also indicated that *circFUT10* and *circLMO7* could regulate myogenesis.[27,28] Interestingly, *circZNF609* is an endogenous circRNA that may be associated with polyribosomes for translation, thus promoting myoblast proliferation.[29] Overall, additional study on the regulation by circRNAs of bovine skeletal muscle development is of great significance for the beef production industry.

To explore the role of circRNAs in bovine skeletal muscle development, we obtained and analyzed the circRNA sequencing data of bovine muscle tissue from NCBI: GSE87908 (https://www.ncbi.nlm.nih.gov/geo/query/acc.cgi?token=atglausktpsjlel&acc=GSE87908).

Received 12 July 2019; accepted 17 October 2019;
https://doi.org/10.1016/j.omtn.2019.10.019.

[2]These authors contributed equally to this work.

Correspondence: Hong Chen, Shaanxi Key Laboratory of Animal Genetics, Breeding and Reproduction, College of Animal Science and Technology, Northwest A&F University, Shaanxi 712100, China.
E-mail: chenhong1212@263.net

 © 2019 The Authors.
This is an open access article under the CC BY license (http://creativecommons.org/licenses/by/4.0/).

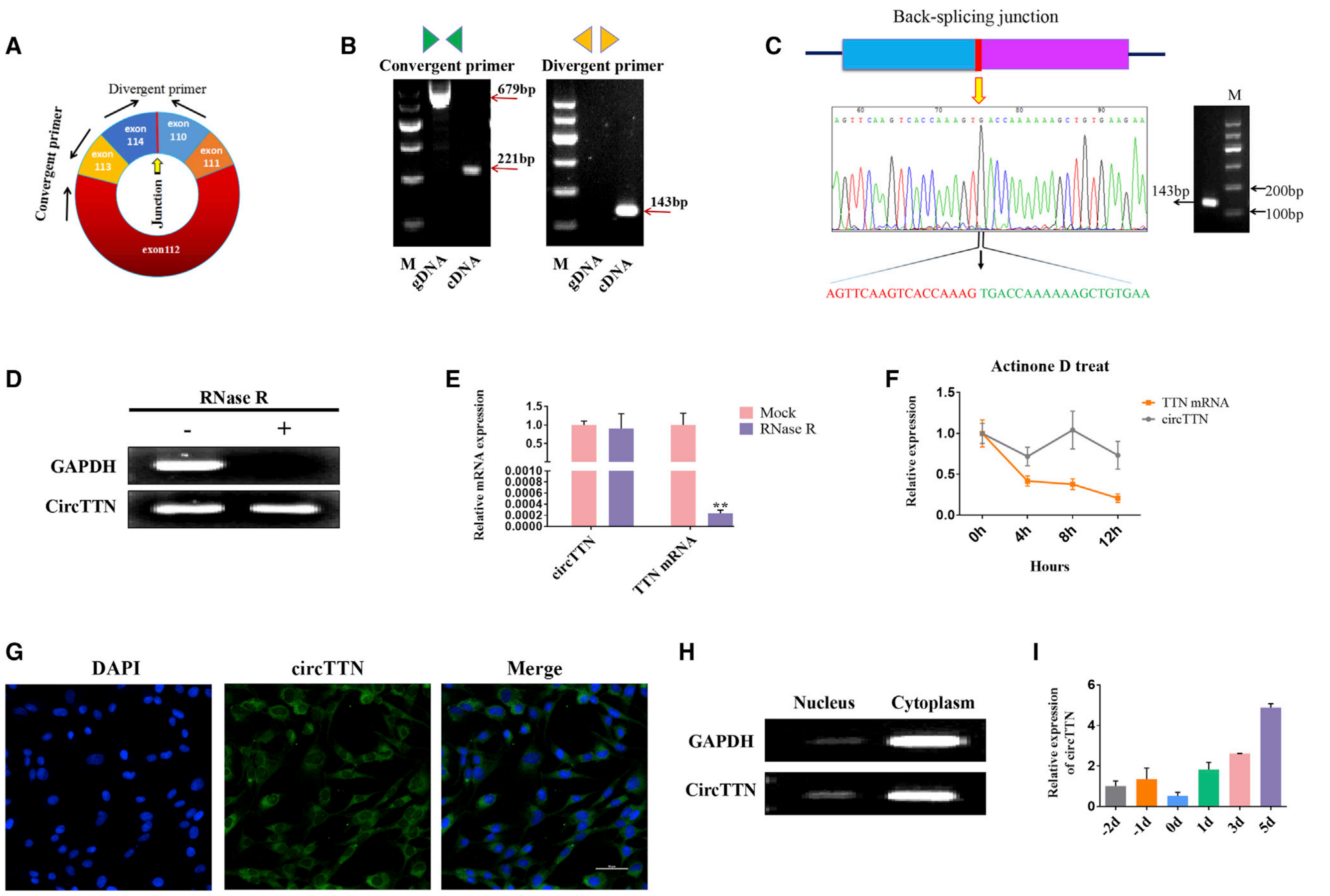

Figure 1. Characterization of Bovine *circTTN*

(A) Schematic view illustrating the design of primers for *circTTN*. A divergent primer was also used in quantitative real-time PCR. (B) A convergent primer and divergent primer were used to confirm the circular nature of *circTTN*. (C) The circular junction of *circTTN* was identified by using a divergent primer on Sanger sequencing. (D) RNase R detected the presence of *circTTN*. (E) The expression of *circTTN* and *TTN* mRNA in myoblasts treated with RNase R was determined by quantitative real-time PCR. (F) Quantitative real-time PCR for the abundance of *circTTN* and *TTN* mRNA in bovine primary myoblasts treated with Actinomycin D at the indicated time points. (G) RNA-FISH assay was performed to determine *circTTN* subcellular localization. Blue indicates nuclei stained with DAPI; green indicates the RNA probe that recognizes *circTTN*. Scale bar, 50 μm. (H) The expression of *circTTN* in the cytoplasm and nuclear was detected by semiquantitative PCR. (I) The expression of *circTTN* in myoblasts differentiated for −2, −1, 0, 1, 3, and 5 days is shown. Data are presented as means ± SEM for three individuals.

We noticed that *circTitin* (*circTTN*) was differentially expressed in fetal and adult bovine muscle tissues and selected it as a candidate circRNA. The full length of *circTTN* was 675 nt and was named after its host gene *Titin* (*TTN*), which is located on chromosome 2 (Text S1). Our results showed that *circTTN* is able to contribute dramatically to bovine primary myoblast proliferation and differentiation. Further examination revealed that *circTTN* acts as a sponge of miR-432 and activates the insulin-like growth factor 2 (*IGF2*)/phosphatidylinositol 3-kinase (*PI3K*)/*AKT* signaling pathway. Our research may provide new insights into complex RNA regulation with implications for the beef cattle industry in China.

RESULTS

Characterization of Bovine *circTTN*

To confirm the circular nature of *circTTN*, we designed two pairs of primers, oriented in the divergent direction and in the convergent direction (Figure 1A), and then used amplified cDNA (RNase R treated) or genomic DNA (gDNA) as templates for amplification. The results showed that the divergent primers amplified the expected band from the cDNA but not from the gDNA. With the use of the convergent primers, products were amplified from either the cDNA or the gDNA samples (Figure 1B). We verified the putative *circTTN* junction by Sanger sequencing (Figure 1C). The results were consistent with the sequencing data. After treatment with RNase R, we found there was no significant decrease in *circTTN* expression, but the expression levels of *TTN* and glyceraldehyde 3-phosphate dehydrogenase (*GAPDH*) mRNA were reduced (Figures 1D and 1E). We then investigated the stability of *circTTN* in bovine primary myoblasts. Total RNA was harvested at the indicated time points after treatment with Actinomycin D. Analysis of *circTTN* and *TTN* mRNA revealed that the *circTTN* was highly stable, with a transcript half-life exceeding 12 h, whereas the associated linear transcript

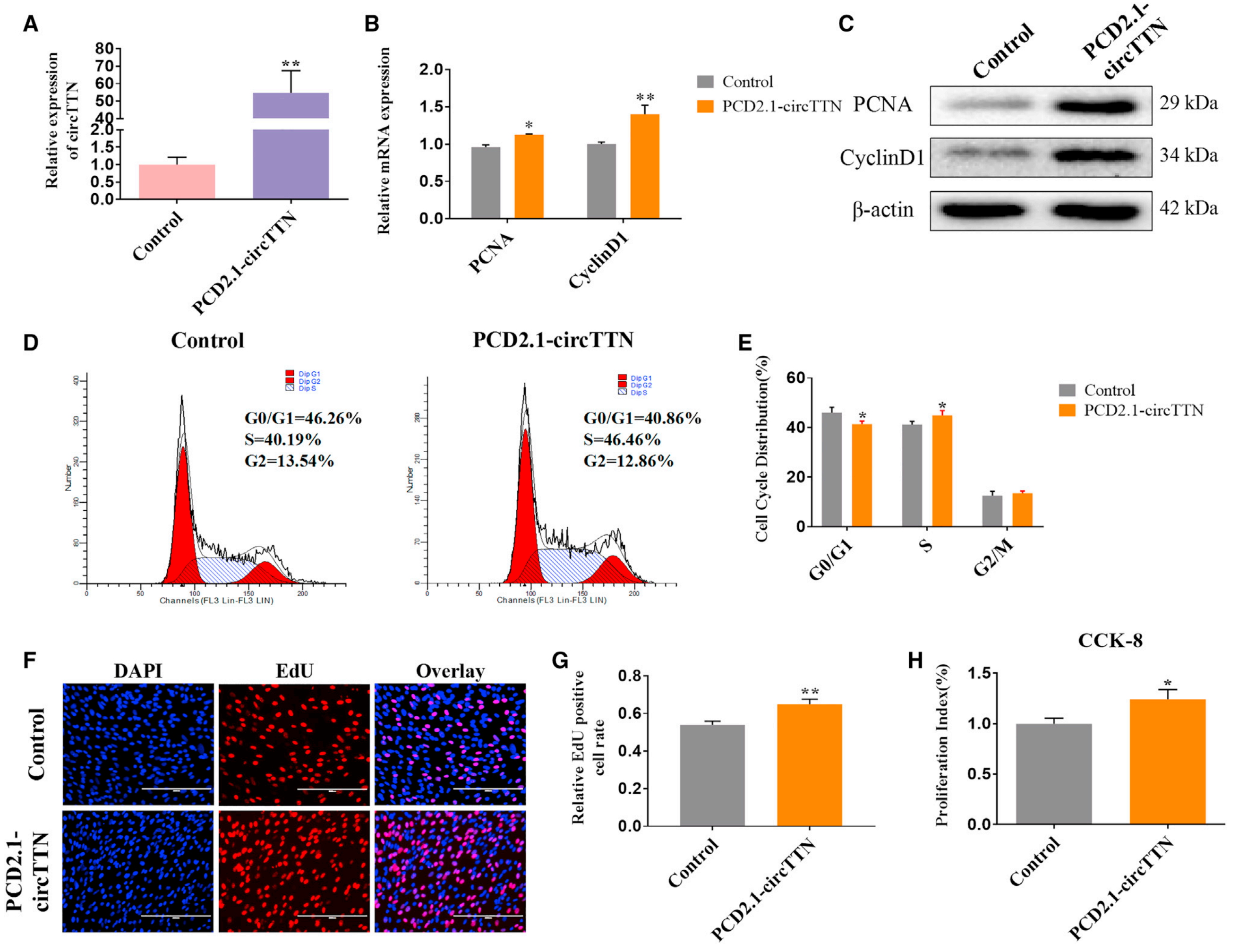

Figure 2. Effect of *circTTN* Overexpression on Bovine Primary Myoblast Proliferation

(A) Visualization of the efficiency of the *circTTN* overexpression vector PCD2.1-*circTTN* by quantitative real-time PCR. (B) Detection of the expression levels of the cell proliferation genes proliferating cell nuclear antigen (*PCNA*) and *Cyclin D1* mRNA by quantitative real-time PCR. (C) Detection of PCNA and Cyclin D1 protein expression levels by western blot analysis, β-actin as an internal control gene. (D and E) Bovine primary myoblasts were transfected with PCD2.1-*circTTN*, and cell phases were analyzed by flow cytometry (D) and counted (E). (F and G) The number of 5-ethynyl-2′-deoxyuridine (EdU)-positive bovine myoblasts was detected by EdU after transfection of PCD2.1-*circTTN* (F) and counted using ImageJ (G). EdU staining (red) for positive cells. DAPI staining (blue) for the cell nuclei. Scale bars indicate 200 μm. (H) Cell proliferation index was detected by the cell counting kit-8 (CCK-8) assay. Data are presented as means ± SEM for three individuals. *p < 0.05; **p < 0.01.

exhibited a half-life of <4 h (Figure 1F). To investigate the cellular localization of *circTTN*, we performed RNA fluorescent *in situ* hybridization (RNA-FISH) assay with an RNA probe that specifically recognizes the back-splicing junction region of *circTTN* to determine its subcellular localization (Figure 1G). We also detected the expression of *circTTN* in the nucleus and cytoplasm by semiquantitative PCR and nucleoplasmic separation (Figure 1H). These two results both suggested that *circTTN* is mainly localized in the cytoplasm, suggesting that *circTTN* may regulate gene expression at the post-transcriptional level. We found that *circTTN* is generally expressed in various fetal (Figure S5A) and adult cattle tissues (Figure S5B) but showed upregulated expression in fetal, calf, and adult bovine muscle tissue (Figure S5C). The expression of *circTTN* was higher in the differentiation period compared with the level during the proliferation period and was also upregulated during myoblast differentiation (Figure 1I). Taken together, our results suggested that *circTTN* may be a positive regulating and stable circRNA for muscle development.

Effect of *circTTN* on Bovine Primary Myoblast Proliferation

To determine the role of *circTTN* in the proliferation of bovine primary myoblasts, we transfected PCD2.1-*circTTN* into myoblasts to overexpress *circTTN* significantly (Figure 2A). To assess its function, we used cell counting kit-8 (CCK-8), 5-ethynyl-2′-deoxyuridine (EdU), flow cytometry, quantitative real-time PCR, and western

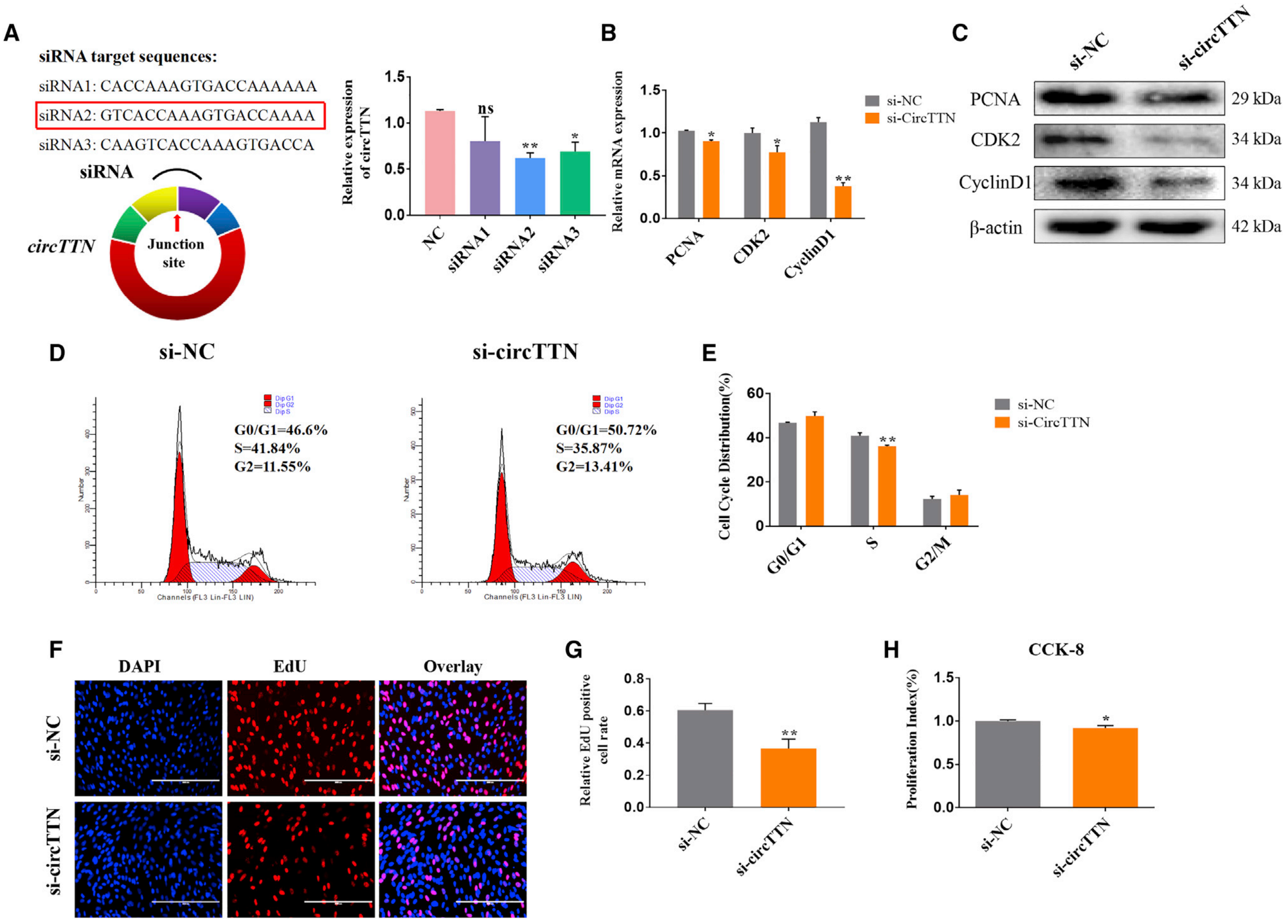

Figure 3. Effect of *circTTN* Knockdown on Bovine Primary Myoblast Proliferation

(A) The interference efficiency of the *circTTN* siRNAs (si-*circTTN*) was detected by quantitative real-time PCR. (B) Detection of the expression levels of the cell proliferation genes *PCNA*, *CDK2*, and *CyclinD1* mRNA by quantitative real-time PCR. (C) Detection of PCNA, CDK2, and Cyclin D1 protein expression levels by western blot analysis, β-actin acts as an internal control gene. (D and E) Bovine primary myoblasts were transfected with si-*circTTN*, and cell phases were analyzed by flow cytometry (D) and counted (E). (F and G) The number of EdU-positive bovine primary myoblasts was detected by EdU after transfection of si-*circTTN* (F) and counted using ImageJ (G). EdU staining (red) for positive cells. DAPI staining (blue) for the cell nuclei. Scale bars indicate 200 μm. (H) Cell proliferation index was detected by cell counting kit-8 (CCK-8) assay. Data are presented as means ± SEM for three individuals. $^*p < 0.05$; $^{**}p < 0.01$.

blotting assays. First, we detected the effect of *circTTN* on expression of the cell proliferation-related genes *Cyclin D1* and proliferating cell nuclear antigen (*PCNA*) and found that *circTTN* significantly increased the expression of these genes at both the mRNA and protein levels (Figures 2B and 2C). The cell-cycle analysis revealed that over-expression of *circTTN* increased the proportion of myoblasts in the S phase and decreased the number of cells in the G0/G1 phase (Figures 2D and 2E and S1). The results of the EdU staining also showed a higher number of EdU-positive cells relative to that in the control group (Figures 2F and 2G). Finally, the CCK-8 assay also revealed that *circTTN* expression significantly improved cell viability (Figure 2H).

Next, small interfering RNAs (siRNAs) were designed to target the back-splicing junction of *circTTN*. After bovine primary myoblasts were transfected with siRNA1, siRNA2, and siRNA3, the expression of *circTTN* was detected by quantitative real-time PCR, and we selected the most effective siRNA2 (Figure 3A). We found that the knockdown of *circTTN* significantly decreased the expression of the cell proliferation-related genes *PCNA*, cyclin-dependent kinase 2 (*CDK2*), and *Cyclin D1* at the mRNA and protein levels (Figures 3B and 3C). The cell-cycle analysis revealed that *circTTN* siRNAs (si-*circTTN*) reduced the proportion of myoblasts in the S phase and increased the number of cells in G0/G1 and G2/M phases (Figures 3D and 3E and S2). Additionally, both the CCK-8 assay (Figure 3H) and the EdU-staining positive cell population (Figures 3F and 3G) were lower in the si-circTTN-treated cells compared to those in the control. These results demonstrated that *circTTN* promotes proliferation of bovine primary myoblasts.

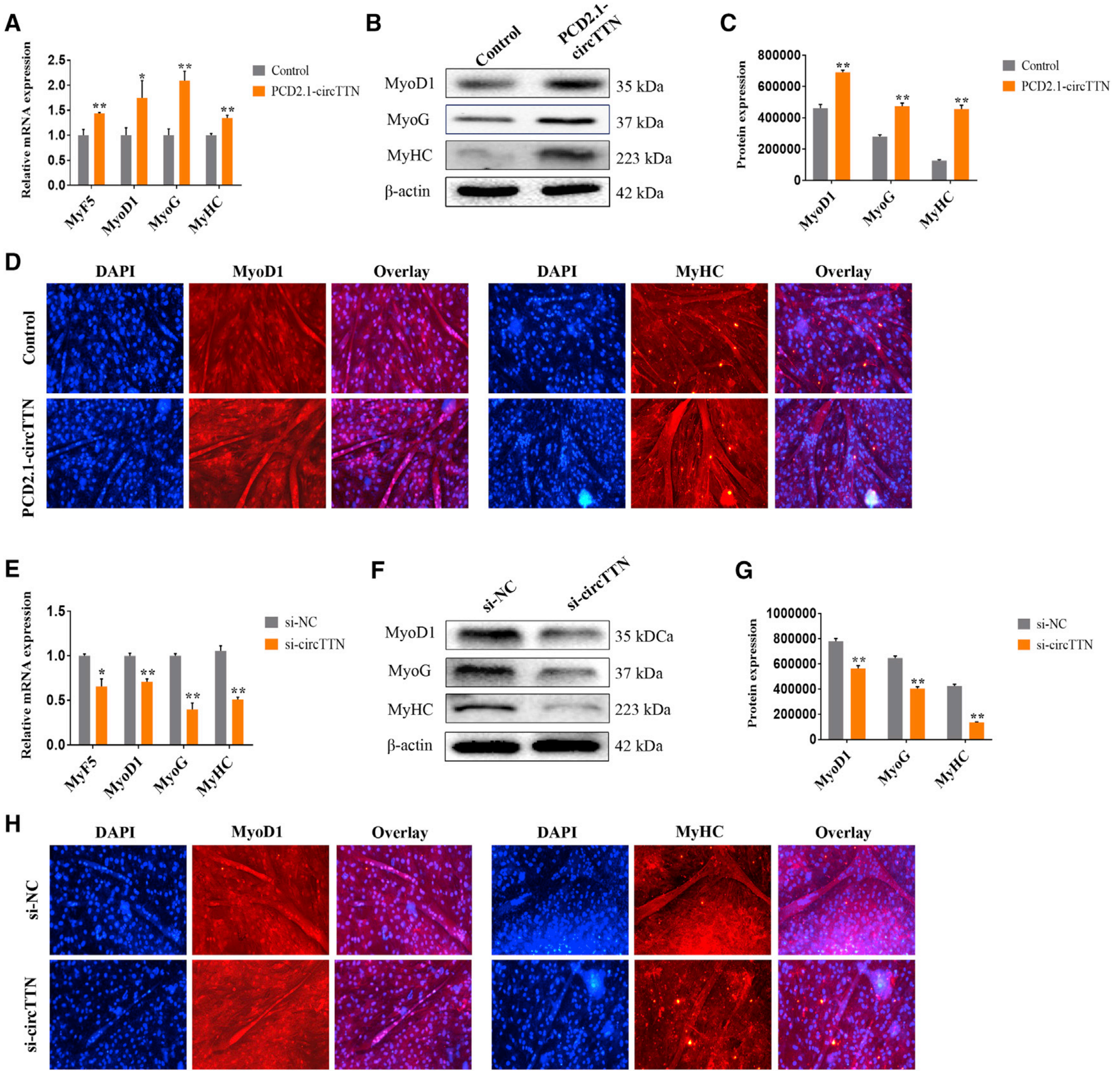

Figure 4. Effect of *circTTN* on Bovine Primary Myoblast Differentiation

(A) Bovine primary myoblasts were transfected with PCD2.1-*circTTN*, and the expression levels of myogenic differentiation marker genes *MyF5*, *MyoD1*, *MyoG*, and *MyHC* mRNA were detected by quantitative real-time PCR. (B and C) Detection of MyoD1, MyoG, and MyHC protein expression levels by western blot analysis (B), and protein band density was also analyzed by ImageJ (C). β-Actin acts as an internal control gene. (D) Cell differentiation was detected by immunofluorescence (*MyoD1* [left] and *MyHC* [right]) and observed under a fluorescence microscope. Scale bar indicates 200 μm. (E) Transfection of the si-*circTTN* and the expression levels of myogenic differentiation marker genes *MyF5*, *MyoD1*, *MyoG*, and *MyHC* mRNA were detected by quantitative real-time PCR. (F and G) MyoD1, MyoG, and MyHC protein levels were detected by western blot analysis (F), and protein band density was also analyzed by ImageJ (G). (H) Cell differentiation was also detected by immunofluorescence (*MyoD1* [left] and *MyHC* [right]) and observed under a fluorescence microscope. Scale bar indicates 200 μm. Data are presented as means ± SEM for three individuals. $^{*}p < 0.05$; $^{**}p < 0.01$.

Effect of *circTTN* on Bovine Primary Myoblast Differentiation

To investigate the involvement of *circTTN* in bovine myoblast differentiation, the expression levels of established myogenic differentiation markers, myogenic factor 5 (*MyF5*), myogenic differentiation 1 (*MyoD1*), myogenin (*MyoG*), and myosin heavy chain (*MyHC*), were detected in primary cattle myoblasts treated with PCD2.1-*circTTN* for 4 days during differentiation. As shown in Figure 4A, the overexpression of *circTTN* efficiently enhanced the expression of *MyF5*, *MyoD1*, *MyoG*, and *MyHC* at the mRNA level (Figure 4A). Moreover, the protein levels of MyoD1, MyoG, and MyHC were also significantly increased (Figures 4B and 4C). In contrast, we used a specific siRNA (si-*circTTN*) targeting *circTTN* to examine the influence of *circTTN* on bovine myoblasts differentiation. The knockdown of *circTTN* significantly suppressed the expression of myogenic markers *MyF5*, *MyoD1*, *MyoG*, and *MyHC* at the mRNA level (Figure 4E), as well as that of MyoD1, MyoG, and MyHC proteins (Figures 4F and 4G). As shown in the immunofluorescence assay results presented in Figures 4D and 4H, circ*TTN* overexpression facilitated

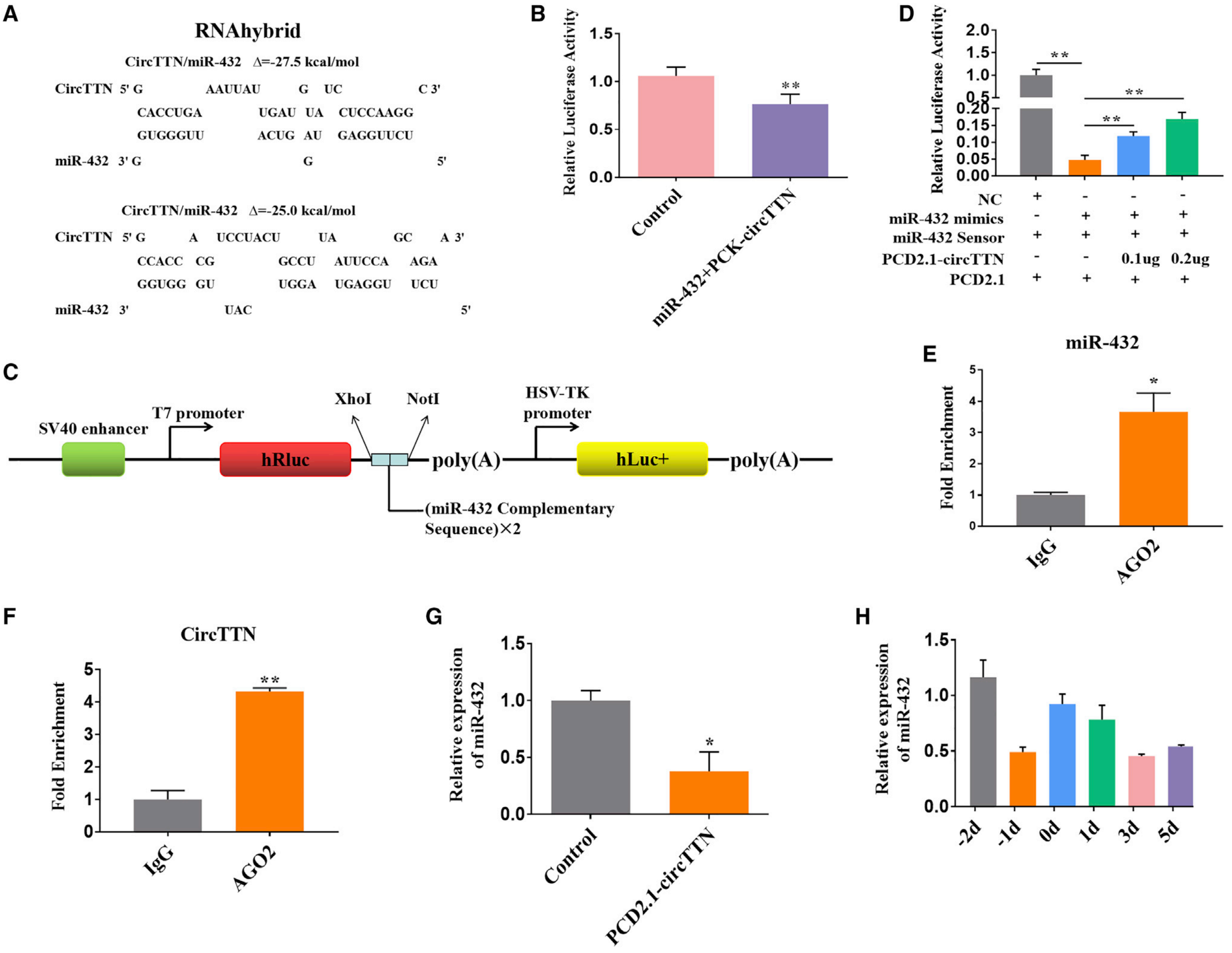

Figure 5. *circTTN* Acts As a Sponge for miR-432

(A) RNAhybrid predicted miR-432 binding sites at two distinct positions in *circTTN*. (B) The miR-432 mimic was cotransfected with PCK-*circTTN* into HEK293T cells. Then, the relative luciferase activity was analyzed 24 h after the transfection. Renilla luciferase activity was normalized to Firefly luciferase activity. (C) Schematic diagram of the miR-432 sensor structure. (D) The miR-432 sensor was cotransfected with the miR-432 mimic and/or PCD-*circTTN* into HEK293T cells. (E and F) Fold enrichment of miR-432 (E) and *circTTN* (F) quantified by quantitative real-time PCR after the RIP assay with Ago2 antibody. IgG was used as the negative control. (G) The expression of miR-432 after overexpression of the *circTTN* was detected by quantitative real-time PCR. (H) The expression of miR-432 in bovine primary myoblasts differentiated for −2, −1, 0, 1, 3, and 5 days is shown. Values are means ± SEM for three individuals. *$p < 0.05$; **$p < 0.01$.

not only the expression of MyoD1 and MyHC but also myotube formation (Figure 4D); however, the knockdown of circ*TTN* reduced expression of MyoD1 and MyHC and myotube formation (Figure 4H). Collectively, these results indicated that *circTTN* acts to promote bovine myoblast differentiation.

circTTN Acts as a Sponge for miR-432

Our results showed that *circTTN* can promote the proliferation and differentiation of bovine myoblasts. Recent work has suggested that different classes of noncoding RNAs may interact with each other, and this interaction regulates their expression.[30] circRNAs are noncoding RNAs that can act as competing endogenous sponges to regulate the expression of miRNAs.[6,12] Thus, we next analyzed the complementarity of the *circTTN* sequence with miRNA using the RNAhybrid bioinformatics program. We found that *circTTN* has perfect target site complementarity with miR-432, and the two binding sites are shown in Figure 5A. The luciferase assay revealed that miR-432 significantly inhibited Renilla luciferase (Rluc) expression of psiCHECK-2-*circTTN* (PCK-*circTTN*) in HEK293T cells (Figure 5B). We also generated a miR-432 sensor by inserting two copies of the miR-432 complementary sequence into the psiCHECK-2 vector (Figure 5C). The result showed that miR-432 dramatically reduced Rluc activity of the miR-432 sensor in HEK293T cells. However, overexpression of the *circTTN* partially restored the reduced

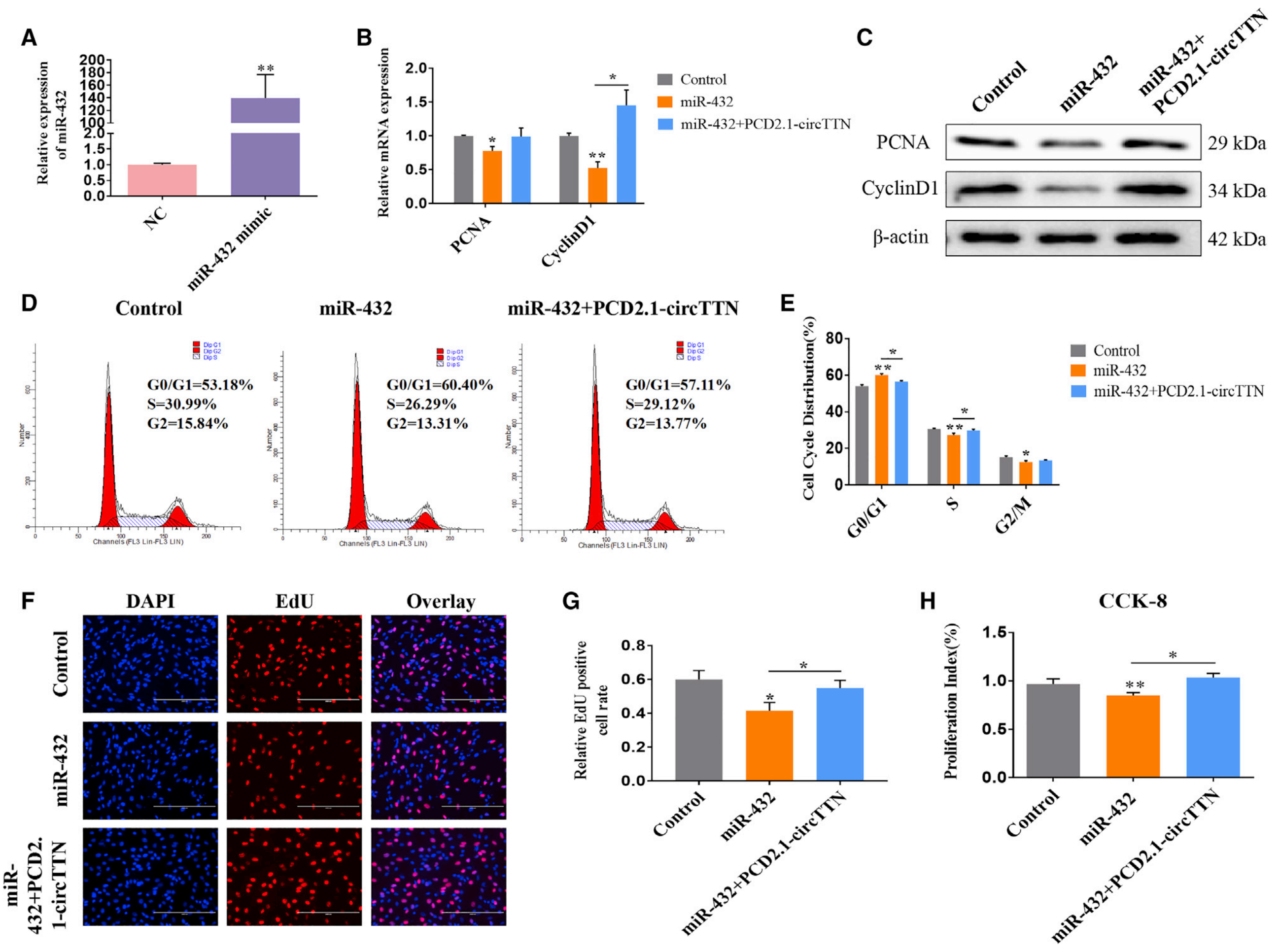

Figure 6. Effect of miR-432 on Proliferation of Bovine Primary Myoblasts

(A) The expression level of miR-432 in bovine primary myoblasts transfected with miR-432 mimic is shown. (B and C) The expression of *PCNA* and *CyclinD1* was detected by quantitative real-time PCR (B) and western blotting (C). β-Actin acts as an internal control gene. (D and E) Bovine primary myoblasts were transfected with miR-432 mimic and/or PCD2.1-*circTTN*, and cell phases were analyzed by flow cytometry (D) and counted (E). (F and G) The number of EdU-positive bovine primary myoblasts was detected by EdU after transfection of miR-432 mimic and/or PCD2.1-*circTTN* (F) and counted using ImageJ (G). EdU staining (red) for positive cells. DAPI staining (blue) for the cell nuclei. Scale bars indicate 200 μm. (H) Cell proliferation index was detected by cell counting kit-8 (CCK-8) assay. Data are presented as means ± SEM for three individuals. $*p < 0.05$; $**p < 0.01$.

Rluc activity induced by binding miR-432 in a dose-dependent manner (Figure 5D). We next performed an RNA immunoprecipitation (RIP) experiment, and quantitative real-time PCR showed successful enrichment of miR-432 (Figure 5E) and *circTTN* (Figure 5F) in the Argonaute 2 (Ago2) pull-down samples compared to the negative control, suggesting that *circTTN* binds to miR-432 via the Ago2 protein. Next, we transfected PCD2.1-*circTTN* into bovine primary myoblasts and significantly decreased the abundance of miR-432 (Figure 5G). There was higher expression of miR-432 during the proliferation period compared with the level in the differentiation period. Additionally, expression of miR-432 was downregulated during myoblast differentiation (Figure 5H). Interestingly, the expression levels of miR-432 and *circTTN* showed the opposite trend. Altogether, these findings indicated that *circTTN* interacts with miR-432.

Effects of miR-432 on Proliferation and Differentiation of Bovine Primary Myoblasts

Next, we elucidated the functional role of miR-432 in proliferation and differentiation in bovine primary myoblasts. For cultured bovine primary myoblasts, we transfected miR-432 mimic into cells and confirmed significant overexpression of miR-432 (Figure 6A). The forced expression of miR-432 efficiently attenuated proliferation marker genes *PCNA* and *Cyclin D1* at the mRNA and proteins levels, and these effects were abolished by overexpression of *circTTN* (Figures 6B and 6C). Cell-cycle analysis revealed that the miR-432 mimic reduced the number of myoblasts in the S and G2/M phases and increased the proportion of cells in the G0/G1 phase, suggesting that miR-432 may inhibit bovine myoblast proliferation (Figures 6D and 6E and S3). Detection of CCK-8 and EdU showed that

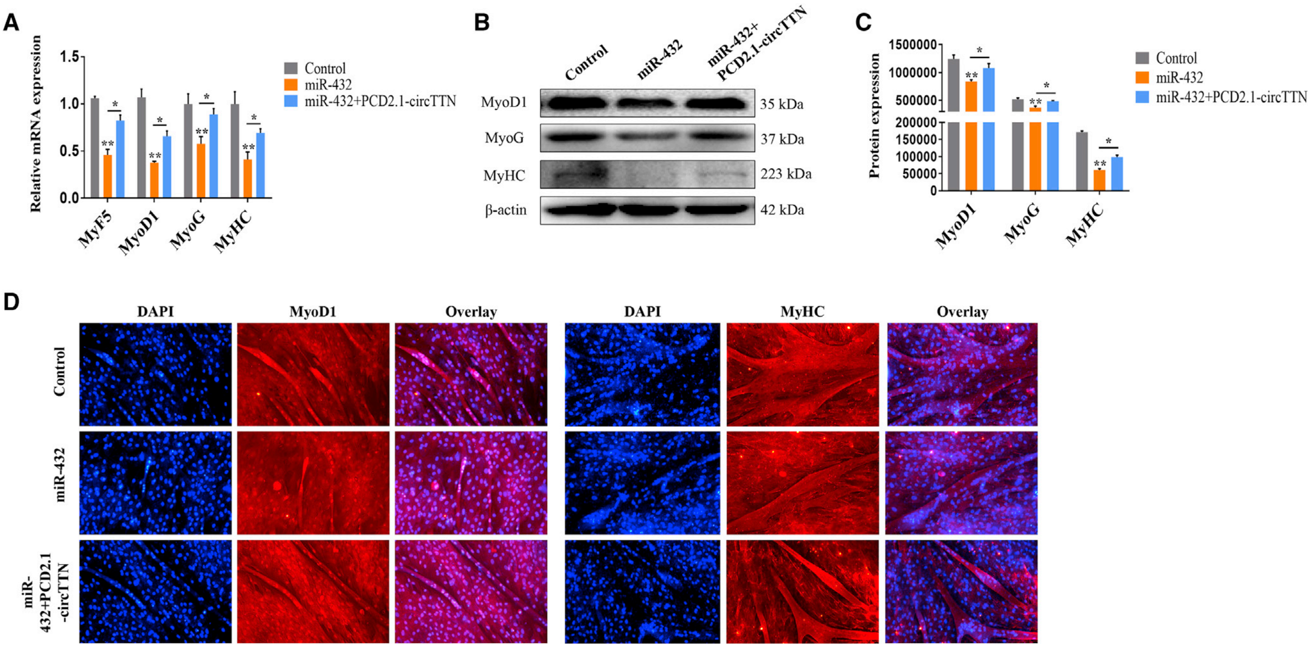

Figure 7. Effect of miR-432 on Differentiation of Bovine Primary Myoblasts

(A) Expression of the myogenic differentiation marker genes *MyF5*, *MyoD1*, *MyoG*, and *MyHC* were detected by quantitative real-time PCR after treated with miR-432 mimic and/or PCD2.1-*circTTN*. (B and C) Detection of MyoD1, MyoG, and MyHC protein expression levels by western blot analysis (B), and protein band density was also analyzed by ImageJ (C). β-Actin acts as an internal control gene. (D) Bovine primary myoblasts were treated with miR-432 mimic and/or PCD2.1-*circTTN*, and cell differentiation was detected by immunofluorescence (*MyoD1* [left] and *MyHC* [right]) and observed under a fluorescence microscope. Scale bar indicates 200 μm. Data are presented as means ± SEM for three individuals. *p < 0.05; **p < 0.01.

overexpression of miR-432 significantly downregulated cell proliferation (Figures 6F–6H). However, we found that cotransfection of miR-432 mimic and PCD2.1-*circTTN* into cattle primary myoblasts resulted in negligible effects on cell proliferation, suggesting that *circTTN* relieved the effect of miR-432 on cell proliferation (Figures 6A–6H).

To assess further the effect of miR-432 on myoblast differentiation, we transfected miR-432 mimic or PCD2.1-*circTTN* into cattle primary myoblasts for 4 days of differentiation. After overexpression of miR-432, the results showed decreased mRNA expression of myogenic differentiation marker genes *MyF5*, *MyoD1*, *MyoG*, and *MyHC* (Figure 7A), with significantly decreased protein levels of MyoD1, MyoG, and MyHC (Figures 7B and 7C). However, cotransfection with PCD2.1-*circTTN* and miR-432 showed somewhat restored expression of *MyF5*, *MyoD1*, *MyoG*, and *MyHC* (Figures 7A–7C). Immunofluorescence assay revealed that miR-432 inhibited MyoD1, MyHC expression, and myotube formation, but this effect was reversed to some extent by *circTTN* overexpression (Figure 7D).

In summary, our above results confirmed that miR-432 could negatively regulate proliferation and differentiation of bovine primary myoblasts, but these effects can be eliminated by overexpression of *circTTN*. Additionally, these results also demonstrated that *circTTN* promotes myogenesis by binding miR-432.

circTTN Serves as a ceRNA for miR-432 to Attenuate Its Inhibitory *IGF2*

To elucidate the potential molecular regulatory mechanism of miR-432 to inhibit proliferation and differentiation of bovine primary myoblasts, we used the bioinformatics software program, TargetScan 7.2. *IGF1* and *IGF2* were considered as two potential target genes of miR-432 (Figure 8A). Next, we constructed *IGF1*-3′ UTR and *IGF2*-3′ UTR (wild-type [WT] and mutant-type [MUT]) luciferase reporter vectors containing potential binding sites of miR-432. The two plasmids were separately cotransfected with miR-432 mimic into HEK293T cells. The luciferase activity of *IGF2*-WT was markedly reduced by miR-432 but had no effect on *IGF2*-MUT (Figure 8B). Moreover, the overexpression of *circTTN* recovered the reduced luciferase activity induced by miR-432 (Figure 8D). Similarly, we found that miR-432 markedly suppressed the expression of *IGF2* at the protein level in proliferation and differentiation stages, but these effects were abrogated by forced expression of *circTTN* (Figures 9C and 9F). Additionally, the luciferase activities of *IGF1*-WT and *IGF1*-MUT were unaffected by miR-432 (Figure 8C). Together, these findings revealed that *circTTN* acts as a bait to alleviate miR-432-mediated inhibition of *IGF2*.

Effects of *IGF2* on Proliferation and Differentiation of Bovine Primary Myoblasts

Next, we used a specific siRNA of *IGF2* (si-*IGF2*) to examine the roles of *IGF2* in myoblast proliferation and differentiation. After

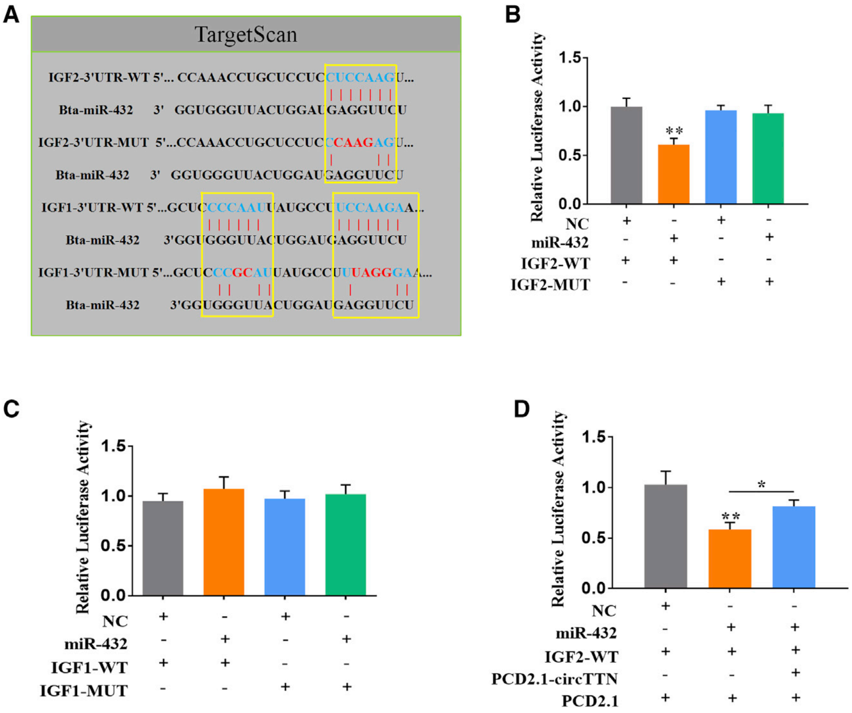

Figure 8. *circTTN* Serves as a ceRNA for miR-432 to Attenuate Its Inhibitory of *IGF2*

(A) TargetScan 7.2 predicted miR-432 binding sites in the *IGF1*-3′ UTR and *IGF2*-3′ UTR. (B) HEK293T cells transfected with the *IGF2*-WT or *IGF2*-MUT was cotransfected with miR-432 mimic or negative control. (C) HEK293T cells transfected with the *IGF1*-WT or *IGF1*-MUT was cotransfected with miR-432 mimic or negative control. (D) HEK293T cells were cotransfected with the miR-432 mimic and *IGF1*-WT or PCD2.1-*circTTN*. The relative luciferase activity was analyzed 24 h after the transfection. Renilla luciferase activity was normalized to the firefly luciferase activity. Values are means ± SEM for three individuals. *$p < 0.05$; **$p < 0.01$.

transfection with si-*IGF2* for myoblasts, the *IGF2* mRNA and protein expression levels were significantly reduced (Figures S5A and S5C and S6B). We also detected the expression of *PCNA*, *CDK2*, and *CyclinD1* by quantitative real-time PCR and western blot assays and found that the knockdown of *IGF2* efficiently attenuated the expression of these genes Figures S5B and S5C. Cell-cycle analysis revealed that si-*IGF2* reduced the proportion of myoblasts in the S and G2/M phase and increased the number of cells in the G0/G1 phase (Figures S4 and S5D and S5E). Moreover, the results of the EdU-staining positive cell population (Figures S5F and S5G) and CCK-8 assay (Figure S5H) were lower than those of the control.

The suppression of *IGF2* also significantly reduced the expression of myogenic differentiation markers *MyF5*, *MyoD1*, *MyoG*, and *MyHC* at the mRNA level (Figure S6A). Additionally, the expression of *MyoD1*, *MyoG*, and *MyHC* was significantly decreased at the protein level (Figures S6B and S6C). Consistently, similar results were obtained by immunofluorescence assay (Figure S6D). Taken together, these data suggested that *IGF2* promotes cattle myoblast proliferation and differentiation.

circTTN Regulates the Activity of the *IGF2/PI3K/AKT* Signaling Pathway

Previous studies had shown the essentiality of *IGF2* for muscle growth and development.[31] In addition, *IGF2* has closely related to the *PI3K/AKT* signaling pathway.[32] Therefore, we hypothesized that *circTTN* may promote proliferation and differentiation of bovine myoblasts through the *IGF2/PI3K/AKT* signaling pathway. To test our hypothesis, western blot analysis was performed to analyze the changes of relevant gene (*IGF2*, *IRS1*, *PI3K*, *PDK1*, *AKT*) levels in the *IGF2/PI3K/AKT* pathway during the proliferation and differentiation phases. For the proliferation stage, the forced expression of *circTTN* remarkably increased the expression of *IGF2*, *IRS1*, *PI3K*, *PDK1*, and *AKT*, whereas the knockdown of *circTTN* decreased the level of *IGF2*, *IRS1*, *PI3K*, *PDK1*, and *AKT* (Figures 9A and 9B). To determine further whether *circTTN* regulates the *IGF2/PI3K/AKT* pathway by affecting miR-432, we transfected miR-432 mimic or PCD2.1-*circTTN*+miR-432 mimic into bovine primary myoblasts. It was obvious that the overexpression of miR-432 reduced the expression of *IGF2*, *IRS1*, *PI3K*, *PDK1*, and *AKT* at mRNA and protein levels, but *circTTN* overexpression alleviated these effects (Figure 9C). Analogously, we took out the semblable consequence in the differentiation stage (Figures 9D–9F). Overall, these results indicated that *circTTN* regulates proliferation and differentiation of bovine primary myoblasts by the *circTTN*-miR-432-*IGF2/PI3K/AKT* axis (Figure 10).

DISCUSSION

Large numbers of circRNAs exist in the human and animal transcriptome, and more and more evidence suggests that circRNA has important roles in regulating cellular functions.[2,6] *circTTN* might be used as an exogenous molecule to treat muscle-related diseases. Here, we used bovine primary myoblasts as a model to study the role of *circTTN* in muscle growth and development, given the fact that cattle, as large animals, could be used as a model for human disease research. ATP2A1 is the causative gene for pseudomyotonia in Chianina cattle, and this new bovine genetic disease is a suitable large-animal model for human Brody disease.[33] Genome-wide analysis of alternative splicing (AS) in cows found that AS plays a major role in the effects of diseases in both humans and cow and is suitable as a model to investigate more serious diseases, such as spinal muscular atrophy and colon cancer.[34] The bovine parasite *Onchocerca ochengi* is closely related to the pathogenic factor (*O. volvulus*) of human river blindness, sharing the same vector with it.[35] In addition, livestock provides many advantages for the discovery of causative variants influencing complex traits. For species, such as cattle, individual polymorphisms may explain a larger

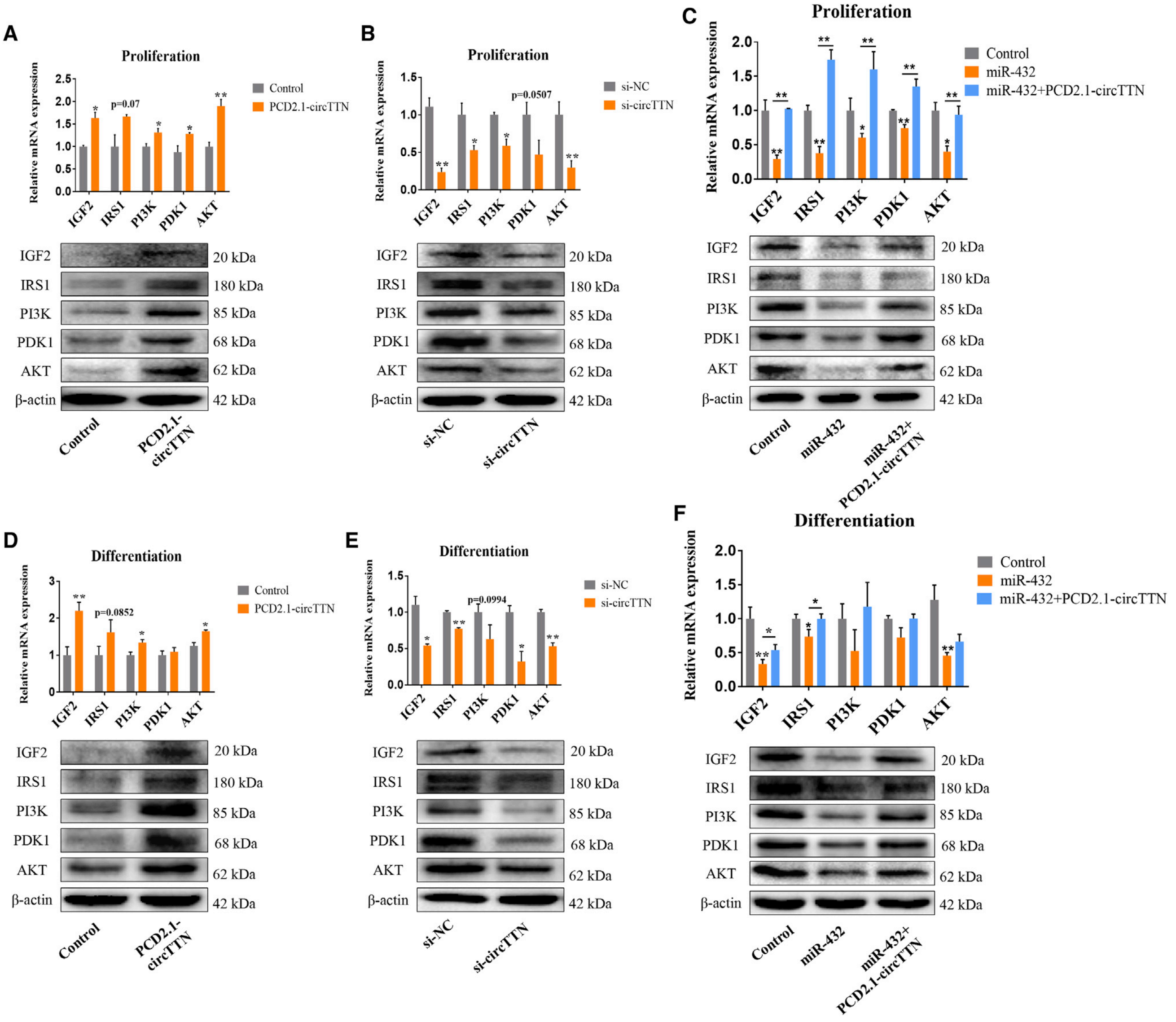

Figure 9. *circTTN* Regulates the Activity of the *IGF2/PI3K/AKT* Signaling Pathway

(A and D) Bovine primary myoblasts transfected with the PCD2.1-*circTTN*, *IGF2/PI3K/AKT* pathway relevant genes (*IGF2*, *IRS1*, *PI3K*, *PDK1*, and *AKT*) were detected by quantitative real-time PCR and western blot analysis in proliferation (A) and differentiation (D) stage. (B and E) Bovine primary myoblasts transfected with si-*circTTN*, *IGF2*, *IRS1*, *PI3K*, *PDK1*, and *AKT* were detected by quantitative real-time PCR and western blot analysis in the proliferation (B) and differentiation (E) stage. (C and F) Bovine primary myoblasts were transfected with miR-432 mimic or PCD2.1-*circTTN* + miR-432 mimic; *IGF2*, *IRS1*, *PI3K*, *PDK1*, and *AKT* were detected by quantitative real-time PCR and western blot analysis in proliferation (C) and differentiation (F) stage. β-Actin acts as an internal control gene. Values are means ± SEM for three individuals. *$p < 0.05$; **$p < 0.01$.

proportion of the variation in these populations compared to the proportion in humans because of intense artificial selection.[36] Overall, cattle can serve as a good model organism for many more human diseases.

To the best of our knowledge, TTN is the largest known protein. It is encoded by 364 exons and plays key structural, developmental, mechanical, and regulatory roles in the heart and skeletal muscle.[37] Mutations in the *TTN* gene have been linked to multiple skeletal and cardiac myopathies.[38] The *circTTN* is derived from five exons (exon 110–exon 114) of the *TTN* gene, and sequencing data reveal that it is differentially expressed between fetal and adult bovine muscle tissues through sequencing data. However, the role of *circTTN* in bovine myogenesis has not been previously described. In this study, we found that *circTTN* could facilitate proliferation and differentiation of bovine primary myoblasts. Mechanistically, we confirmed

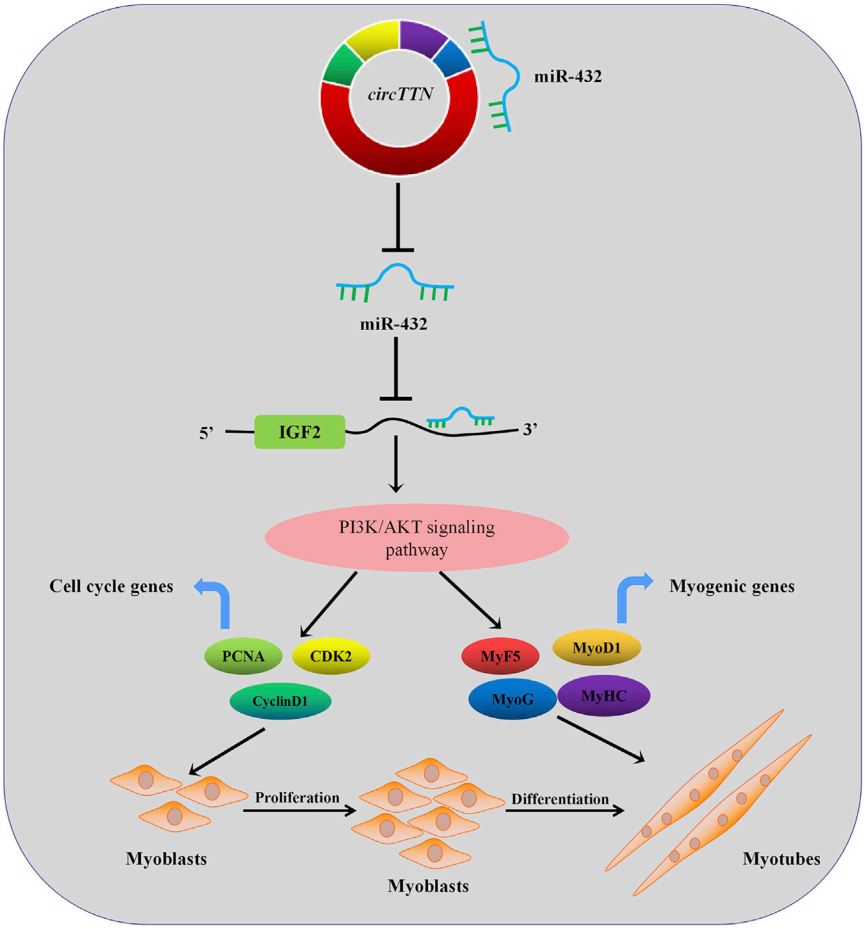

Figure 10. Model of the Action *circTTN* Competitively Sponging miR-432 Mediating Myoblast Proliferation and Differentiation

that *circTTN* acts as a decoy for miR-432 to relieve the miRNA inhibition effect on *IGF2* and to activate further the *IGF2*/*PI3K/AKT* signaling pathway.

Previous work has shown that that circRNA exhibits multiple mechanisms of action, including acting as a ceRNA,[12] acting on RNA transcription,[39] participating in protein translation,[40] and interacting with proteins.[6] The ceRNA hypothesis is a previously unknown pattern for gene expression regulation that was formally proposed in 2011.[41] ceRNAs are transcripts that can regulate each other at the post-transcription level by competing for shared miRNAs.[42] Various types of ceRNA have been found, including mRNA, pseudogene transcripts, lncRNA, and circRNA.[11,43–45] However, circRNAs, as particular endogenous RNAs, have become a new focus among the ceRNA family after lncRNAs. circRNA can act as sponges to block and inhibit miRNAs from binding to their target sites. Here, in order to determine potential downstream effectors of *circTTN*-mediated regulation of myogenesis, we screened miR-432. RNAhybrid prediction analysis showed that *circTTN* includes a perfect target site complementarity with miR-432. miR-432 targeting *E2F3* and *P55PIK* inhibits proliferation and differentiation of myoblasts.[46] In disease, miR-432 inhibits proliferation of lung adenocarcinoma cells by targeting *E2F3* and *ALX*.[47] hsa_circ_0008039 promotes the proliferation and migration of breast cancer cells by regulating the miR-432-5p/*E2F3* axis.[48] Consistent with these results, our experiments demonstrated that miR-432 is a negative regulator of bovine myogenesis. Nevertheless, these effects could be eliminated by overexpression of *circTTN*, suggesting that *circTTN* was reasonable and effective for the sponge effect on miR-432. Thus, our results indicate that *circTTN* could serve as a miR-432 sponge for myogenesis.

Several miRNAs that target *IGF2* have been reported, including miR-223,[49] miR-125b,[50] miR-185,[51] miR-3941,[52] miR-615-3p,[53] and miR-193-5p,[54] etc. Among them, the inhibition of *IGF2* by miR-223 resulted in the repression of myoblast proliferation.[49] Sheep enhanced muscularity transcript lncRNA (lnc-SEMT) modulates *IGF2* expression through sponge miR-125b to promote muscle development and growth in sheep.[50] Finally, miR-193-5p regulates angiogenesis by *IGF2* in type 2 diabetic cardiomyopathy.[54] In this study, *IGF2* was first identified as a direct target gene of miR-432 by luciferase activity and western blot assay. *IGF2* is highly expressed in skeletal muscle and has been previously identified as a quantitative trait locus (QTL) of muscle mass, as change in a single base of pig *IGF2* intron 3 is a major QTL affecting muscle growth, heart size, and fat deposition.[55] During differentiation of, *IGF2* is regulated by mammalian target of rapamycin (mTOR) and by amino acid sufficiency through *IGF2* promoter 3 and a downstream enhance.[56] In yak, *IGF2* promotes fibroblast proliferation by inhibiting the expression of *IGF1R* and *PI3KCG*.[57] The binding of IGFs (*IGF1* and *IGF2*) to their receptors induces autophosphorylation of its tyrosine residues, which in turn, activate a variety of intracellular signaling pathways, including the *PI3K/AKT* and mitogen-activated protein kinase (*MAPK*) pathways that trigger various biological processes, such as cell proliferation, differentiation, and apoptosis.[58] *IGF2* promotes metabolism and proliferation of rainbow trout myocytes by activating *PI3K/AKT* and *MAPK* signaling pathways.[59] In terms of cancer, IGF2 mRNA-binding protein 2 (Imp2) regulates the activity of *IGF2*, which further activates *PI3K/AKT* signaling to promote glioblastoma (GBM) malignancy.[32] Forced *circTTN* expression increased the protein expression of *IGF2* and genes (*IRS1*, *PI3K*, *PDK1*, and *AKT*) involved in the *PI3K/AKT* pathway, but knockdown of *circTTN* has the opposite effect. Furthermore, overexpression of miR-432 significantly inhibited the expression of *IGF2*, *IRS1*, *PI3K*, *PDK1*,

and *AKT*, but these effects were abolished by increased *circTTN*. These results demonstrated that *IGF2*, *IRS1*, *PI3K*, *PDK1*, and *AKT* are downstream molecules of miR-432 in bovine myoblasts.

In summary, this study demonstrated the role and regulatory mechanism of circ*TTN* in bovine primary myoblasts. Our research identified for the first time that *circTTN* regulates proliferation and differentiation of bovine myoblasts by competing with miR-432 as a ceRNA, which activates the *IGF2/PI3K/AKT* signaling pathway. This helps elucidate the molecular events associated with muscle development and muscle disease and unravels the complex molecular mechanism of myogenesis.

MATERIALS AND METHODS

Sample Preparation

Fetal bovines were obtained from a local slaughterhouse in Xi'an, China. Six tissues of Qinchuan cattle at embryonic and adult stage (heart, liver, spleen, lung, kidney, and muscle) were obtained and stored at −80°C in a freezer until use. A total of nine muscle tissue samples of Qinchuan cattle were collected from three developmental states (fetal, 90 days; calf, 6 months; and adult, 24 mouths). Animal samples used in this study were approved by the Animal Care and Use Committee of Northwest A&F University.

RNA Preparation and Quantitative Real-Time PCR

Total RNA samples of cells or tissues were extracted using TRIzol Reagent (Takara, Dalian, China) as the manufacturer's instructions. RNA was reverse transcribed into cDNA using the PrimeScript RT Reagent Kit with gDNA Eraser (Takara, Dalian, China). The nuclear and cytoplasmic fractions were separately extracted using a nucleoplasmic separation kit (PARIS kit; Life Technologies, Carlsbad, CA, USA). For RNase R treatment, 1 μg of total RNA was incubated for 15 min at 37°C with 2 units μg^{-1} of RNase R and subsequently purified using an RNeasy MinElute Cleaning Kit (QIAGEN, Hilden, Germany). The quantitative real-time PCR analyses were performed with the SYBR Green Kit (Genestar, Beijing, China), and quantitative real-time PCR was performed on three biological replicates at each time point. *GAPDH* and *U6* (for miRNA) genes were used as internal controls for normalization of the data. All spanning the distal ends of circRNAs with sequence specificity was detected using Primer-BLAST (NCBI). The relative expression level was calculated by the comparative cycle threshold ($2^{-\Delta\Delta Ct}$) method. The primers in our study are listed in Table S1.

Vector Construction

The whole length of *circTTN* was cloned into the overexpression vector of PCD2.1 using the two times TSINGKE Master Mix (blue) (TSINGKE, Beijing, China) and was designated PCD2.1-*circTTN*. The fragment of the *IGF2* 3′ UTR-WT, including the binding site of miR-432, was amplified and inserted into the psiCHECK-2 vector (*IGF2*-WT) (Promega, Madison, WI, USA) at the 3′ end of the Renilla gene using restriction enzymes *Xho*I and *Not*I (Takara, Dalian, China) and T4 DNA ligase. The mutant type psiCHECK-2-*IGF2*-3′ UTR-MUT (*IGF2*-MUT) was generated by mutating complementary to the seed region of the miR-432 using overlapping PCR. Finally, *IGF1*-WT and *IGF1*-MUT were inserted into the psiCHECK-2 vector in the same way. A miR-432 sensor (with a perfect miR-432 binding site) was generated by insertion of two sequences that are completely complementary to the mature sequence of miR-432 after the psiCHECK-2 vector Rluc. Therefore, it can respond to miR-432 reflecting by the fluorescence activity alteration. Similarly, the vector of PCK-*circTTN* was obtained using the same method. Primer sequences are shown in Table S2, and all constructs were verified by sequencing (Sangon Biotech, Shanghai, China).

Cell Culture and Treatment

Bovine primary myoblasts were isolated and cultured from bovine longissimus muscle as previously described.[60] We plated bovine primary myoblasts at the stage of 80% confluence at a density of 5×10^5 cells per well in six-well plates in 2 mL 20% fetal bovine serum (FBS) culture medium per well or 1×10^5 cells per well in 96-well plates (NEST, Wuxi, China) in 100 μL culture medium per well and incubated them, as described previously.[60] After growth to ∼80% confluence, the cells were transfected with PCD2.1-*circTTN* (2 μg/mL), si-*circTTN* (RiboBio, Guangzhou, China), miR-432 mimics (RiboBio, Guangzhou, China), si-*IGF2* (RiboBio, Guangzhou, China), or miR-432 mimics+PCD2.1-*circTTN* using Lipofectamine 2000 (Invitrogen, USA), respectively. After incubation, the bovine primary myoblasts were used for the different assays outlined below. To induce differentiation of bovine primary myoblasts, the culture medium was changed to high-glucose DMEM with 2% horse serum. Transcription was blocked by adding 1 μM Actinomycin D solution (Leagene, Beijing, China) to the cell culture medium. The si-*circTTN* targets the *circTTN* junction (Figure 3A). The si-*IGF2* target sequence is 5′-TTCTACT TCAGCCGACCAT-3′.

RNA-FISH assay

A RNA-FISH assay was performed in bovine primary myoblasts following the instructions of the probe manufacturer (RiboBio, Guangzhou, China). The probe sequence for circTTN is 5′-AGCTTTTTGG TCACTTTGGTGACTTGAAC-3′. The cells were plated into coverglass in the 6-well plates and were cultured to 70%∼80% confluence and then fixed. After 0.1% Triton X-100 transmembrane treatment, the cells were incubated with 20 mg/mL of circTTN probe overnight at 37°C. Nuclei were counterstained with DAPI. Images were acquired by using an FV1200 laser confocal microscope (Olympus).

Flow Cytometry for the Cell Cycle

Cell-cycle assay kits were used to analyze cell cycles in different treatment groups (Multisciences, Hangzhou, China). We cultured bovine primary myoblasts in 6-well plates (1×10^6 cells/well) with 2 mL culture medium. We collected cells and washed them once with PBS buffer after treatment for 24 h. Next, 1 mL of DNA strain solution and 10 μL of permeabilization solution were added to the resuspended cells. Flow cytometry was next performed using the cell suspensions after incubation for 30 min in the dark at room temperature (FACSCanto II; BD Biosciences, USA). Each treatment group had three independent replicates.

CCK-8 Assay

The cells were plated into 96-well culture plates in 100 μL of culture medium per well, and each treatment group had seven independent replicates. After 24 h of incubation at 37°C, 10 μL of CCK-8 reagent (Tiandz, Beijing, China) was added to each well and incubated at 37°C for 4 h. The absorbance of each sample was detected at 450 nm using an enzyme standard instrument (Molecular Devices, Sunnyvale, CA, USA).

EdU Assay

The proliferation circumstances of bovine primary myoblasts were probed by Cell-Light EdU Apollo 567 *In Vitro* Imaging Kit (RiboBio, Guangzhou, China), according to the manufacturer's instructions, and each treatment group had three independent replicates.

Luciferase Activity Assay

HEK293T cells were used to validate interactions and were cultured in DMEM with 10% FBS. When the cell confluence reached about 80%, the miR-432 mimics and PCK-*circTTN* or miR-432 sensor were co-transfected into HEK293T cells using Lipofectamine 2000. Similarly, the miR-432 mimics and *IGF2*-WT or *IGF2*-MUT were cotransfected into cells. In addition, the miR-432 mimics, PCD2.1-*circTTN*, and *IGF2*-WT were cotransfected into cells. After incubation for 24 h, the cells were washed with PBS and collected using 100 μL passive lysis buffer (PLB). Dual-Luciferase Reporter (DLR) Assay System Kit (Promega, Madison, WI, USA) was performed to analyze firefly luciferase activity and Rluc activity, according to the manufacturer's instructions. The firefly luciferase activity was used as a control to normalize the signal value.

RIP

The Magna RIP RNA-Binding Protein Immunoprecipitation Kit was used to perform the RIP assay (Millipore, Bedford, MA, USA), following the manufacturer's protocol. Bovine primary myoblasts at the stage of 80% confluence were collected and lysed using RIP lysis buffer. Then, Ago2 immunoprecipitation was performed using an anti-Ago2 antibody (Abcam, Cambridge, UK), and an immunoglobulin G (IgG) antibody was used as a negative control. Finally, the immunoprecipitated RNA was isolated, and the abundance of *circTTN* and miR-432 in bound fractions was evaluated by quantitative real-time PCR analysis.

Western Blotting Analysis

Total proteins of bovine primary myoblasts were extracted from the different treatment groups using the protein lysis buffer radioimmunoprecipitation assay (RIPA) containing 1% PMSF (Solarbio, Beijing, China). The protein concentration was determined by a bicinchoninic acid kit (Beyotime, Shanghai, China). The collected proteins were boiled with 25% five times protein loading buffer (2-mercaptoethanol [2-ME]) (HAT, Xi'An, China) at 98°C for 10 min, and 20∼40 μg total protein was loaded and separated by SDS-PAGE and then transferred to a polyvinylidene fluoride (PVDF) membrane, blocked in 5% skim milk for about 2 h at room temperature, and incubated with primary antibodies diluted in Primary Antibody Dilution Buffer (HAT, Xi'An, China) overnight at 4°C: anti-β-actin (1:1,000; KM9001T; SungeneBiotech, Tianjin, China); anti-PCNA (1:1,000; D220014; SangonBiotech, Shanghai, China); anti-CyclinD1 (WL01435a), anti-CDK2 (WL02028), anti-IGF2 (WL02665), anti-IRS1 (WL03123), anti-PI3K (WL02240), anti-PDK1 (WL00707), and anti-AKT (WL0003b) (1:500; Wanleibio, Shenyang, China); anti-MyoD1 (ab16148) and anti-MyoG (ab124800) (1;1,000; Abcam, Cambridge, UK); anti-heavy chain cardiac myosin (1:1,000; MyHC, GTX20015; GeneTex, USA). After that, we washed the PVDF membranes with Tris-buffered saline Tween 20 (TBST) buffer and then incubated with horseradish peroxidase (HRP)-conjugated secondary antibody for 1.5 h at room temperature. The HRP-conjugated secondary antibodies were as follows: goat anti-mouse IgG HRP (M21001S; Abmart, Shanghai, China); goat anti-rabbit IgG (H+L)-HRP conjugate (BA1054; Boster, Wuhan, China). Finally, antibody-reacting bands were detected using ECL Super Sensitive Kit (DiNing, Beijing, China).

Immunofluorescence Staining

Bovine primary myoblasts were differentiated for 4 days and then were washed three times with PBS buffer (pH 7.4), followed by fixation with 4% paraformaldehyde for 30 min. Then, cells were permeabilized for 15 min in PBS containing 0.5% Triton X-100. Cells were stained overnight at 4°C using MyoD1 antibody (1;500; ab16148; Abcam, Cambridge, UK) and MyHC antibody (1:250; GTX20015; GeneTex, USA), diluted in 1% BSA. We then incubated them at room temperature for 2 h with the corresponding secondary antibody goat anti-mouse IgG (heavy chain and light chain [H&L])-Alexa Fluor 594 (1:500; RS3608; Immunoway, USA), diluted in 1% BSA in PBS. Nuclei were visualized by DAPI stain. Finally, we washed them three times with PBS and observed under a fluorescence microscope (DM5000B; Leica, Germany).

Statistical Analyses

The experimental results were expressed as the mean ± SEM. All data in this study were analyzed by t tests using GraphPad Prism 7.0 software. $p < 0.05$ was considered statistically significant among means, and $p < 0.01$ was considered highly significant.

SUPPLEMENTAL INFORMATION

Supplemental Information can be found online at https://doi.org/10.1016/j.omtn.2019.10.019.

AUTHOR CONTRIBUTIONS

X.W. and H.C. designed and coordinated the project. X.W. performed the experiments and drafted the manuscript. X.C. modified the manuscript and provided valuable advice. D.D., X.S., J.C., R.J., Z.Y., and S.P. helped perform the experiments and analyzed the data. X.L. and C.L. provided suggestions to the experiments. Y.H. and I.E.E. helped collect tissue sample.

CONFLICTS OF INTEREST

The authors declare no competing interests.

ACKNOWLEDGMENTS

This work was supported by the National Natural Science Foundation of China (no. 31772574).

REFERENCES

1. Rong, D., Sun, H., Li, Z., Liu, S., Dong, C., Fu, K., Tang, W., and Cao, H. (2017). An emerging function of circRNA-miRNAs-mRNA axis in human diseases. Oncotarget *8*, 73271–73281.
2. Zheng, Q., Bao, C., Guo, W., Li, S., Chen, J., Chen, B., Luo, Y., Lyu, D., Li, Y., Shi, G., et al. (2016). Circular RNA profiling reveals an abundant circHIPK3 that regulates cell growth by sponging multiple miRNAs. Nat. Commun. *7*, 11215.
3. Nigro, J.M., Cho, K.R., Fearon, E.R., Kern, S.E., Ruppert, J.M., Oliner, J.D., Kinzler, K.W., and Vogelstein, B. (1991). Scrambled exons. Cell *64*, 607–613.
4. Jeck, W.R., and Sharpless, N.E. (2014). Detecting and characterizing circular RNAs. Nat. Biotechnol. *32*, 453–461.
5. Salzman, J., Gawad, C., Wang, P.L., Lacayo, N., and Brown, P.O. (2012). Circular RNAs are the predominant transcript isoform from hundreds of human genes in diverse cell types. PLoS ONE *7*, e30733.
6. Memczak, S., Jens, M., Elefsinioti, A., Torti, F., Krueger, J., Rybak, A., Maier, L., Mackowiak, S.D., Gregersen, L.H., Munschauer, M., et al. (2013). Circular RNAs are a large class of animal RNAs with regulatory potency. Nature *495*, 333–338.
7. Guo, J.U., Agarwal, V., Guo, H., and Bartel, D.P. (2014). Expanded identification and characterization of mammalian circular RNAs. Genome Biol. *15*, 409.
8. Glažar, P., Papavasileiou, P., and Rajewsky, N. (2014). circBase: a database for circular RNAs. RNA *20*, 1666–1670.
9. Rybak-Wolf, A., Stottmeister, C., Glažar, P., Jens, M., Pino, N., Giusti, S., Hanan, M., Behm, M., Bartok, O., Ashwal-Fluss, R., et al. (2015). Circular RNAs in the Mammalian Brain Are Highly Abundant, Conserved, and Dynamically Expressed. Mol. Cell *58*, 870–885.
10. Kim, H.H., Kuwano, Y., Srikantan, S., Lee, E.K., Martindale, J.L., and Gorospe, M. (2009). HuR recruits let-7/RISC to repress c-Myc expression. Genes Dev. *23*, 1743–1748.
11. Zhong, Y., Du, Y., Yang, X., Mo, Y., Fan, C., Xiong, F., Ren, D., Ye, X., Li, C., Wang, Y., et al. (2018). Circular RNAs function as ceRNAs to regulate and control human cancer progression. Mol. Cancer *17*, 79.
12. Hansen, T.B., Jensen, T.I., Clausen, B.H., Bramsen, J.B., Finsen, B., Damgaard, C.K., and Kjems, J. (2013). Natural RNA circles function as efficient microRNA sponges. Nature *495*, 384–388.
13. Hentze, M.W., and Preiss, T. (2013). Circular RNAs: splicing's enigma variations. EMBO J. *32*, 923–925.
14. Li, Z., Huang, C., Bao, C., Chen, L., Lin, M., Wang, X., Zhong, G., Yu, B., Hu, W., Dai, L., et al. (2015). Exon-intron circular RNAs regulate transcription in the nucleus. Nat. Struct. Mol. Biol. *22*, 256–264.
15. Das, A., Das, A., Das, D., Abdelmohsen, K., and Panda, A.C. (2019). Circular RNAs in myogenesis. Biochim. Biophys. Acta. Gene Regul. Mech. Published online April 1, 2019. https://doi.org/10.1016/j.bbagrm.2019.02.011.
16. Frontera, W.R., and Ochala, J. (2015). Skeletal muscle: a brief review of structure and function. Calcif. Tissue Int. *96*, 183–195.
17. Okazaki, K., and Holtzer, H. (1966). Myogenesis: fusion, myosin synthesis, and the mitotic cycle. Proc. Natl. Acad. Sci. USA *56*, 1484–1490.
18. Bentzinger, C.F., Wang, Y.X., and Rudnicki, M.A. (2012). Building muscle: molecular regulation of myogenesis. Cold Spring Harb. Perspect. Biol. *4*, a008342.
19. Chargé, S.B.P., and Rudnicki, M.A. (2004). Cellular and molecular regulation of muscle regeneration. Physiol. Rev. *84*, 209–238.
20. Song, C., Yang, Z., Dong, D., Xu, J., Wang, J., Li, H., Huang, Y., Lan, X., Lei, C., Ma, Y., and Chen, H. (2019). miR-483 inhibits bovine myoblast cell proliferation and differentiation via IGF1/PI3K/AKT signal pathway. J. Cell. Physiol. *234*, 9839–9848.
21. Sun, J., Li, M., Li, Z., Xue, J., Lan, X., Zhang, C., Lei, C., and Chen, H. (2013). Identification and profiling of conserved and novel microRNAs from Chinese Qinchuan bovine longissimus thoracis. BMC Genomics *14*, 42.
22. Li, H., Yang, J., Jiang, R., Wei, X., Song, C., Huang, Y., Lan, X., Lei, C., Ma, Y., Hu, L., and Chen, H. (2018). Long Non-coding RNA Profiling Reveals an Abundant MDNCR that Promotes Differentiation of Myoblasts by Sponging miR-133a. Mol. Ther. Nucleic Acids *12*, 610–625.
23. Zhou, L., Sun, K., Zhao, Y., Zhang, S., Wang, X., Li, Y., Lu, L., Chen, X., Chen, F., Bao, X., et al. (2015). Linc-YY1 promotes myogenic differentiation and muscle regeneration through an interaction with the transcription factor YY1. Nat. Commun. *6*, 10026.
24. Wang, Y., Li, M., Wang, Y., Liu, J., Zhang, M., Fang, X., Chen, H., and Zhang, C. (2019). A Zfp609 circular RNA regulates myoblast differentiation by sponging miR-194-5p. Int. J. Biol. Macromol. *121*, 1308–1313.
25. Li, H., Wei, X., Yang, J., Dong, D., Hao, D., Huang, Y., Lan, X., Plath, M., Lei, C., Ma, Y., et al. (2018). circFGFR4 Promotes Differentiation of Myoblasts via Binding miR-107 to Relieve Its Inhibition of Wnt3a. Mol. Ther. Nucleic Acids *11*, 272–283.
26. Ouyang, H., Chen, X., Li, W., Li, Z., Nie, Q., and Zhang, X. (2018). Circular RNA *circSVIL* Promotes Myoblast Proliferation and Differentiation by Sponging miR-203 in Chicken. Front. Genet. *9*, 172.
27. Wei, X., Li, H., Yang, J., Hao, D., Dong, D., Huang, Y., Lan, X., Plath, M., Lei, C., Lin, F., et al. (2017). Circular RNA profiling reveals an abundant circLMO7 that regulates myoblasts differentiation and survival by sponging miR-378a-3p. Cell Death Dis. *8*, e3153.
28. Li, H., Yang, J., Wei, X., Song, C., Dong, D., Huang, Y., Lan, X., Plath, M., Lei, C., Ma, Y., et al. (2018). CircFUT10 reduces proliferation and facilitates differentiation of myoblasts by sponging miR-133a. J. Cell. Physiol. *233*, 4643–4651.
29. Legnini, I., Di Timoteo, G., Rossi, F., Morlando, M., Briganti, F., Sthandier, O., Fatica, A., Santini, T., Andronache, A., Wade, M., et al. (2017). Circ-ZNF609 Is a Circular RNA that Can Be Translated and Functions in Myogenesis. Mol. Cell *66*, 22–37.e9.
30. Wang, K., Gan, T.Y., Li, N., Liu, C.Y., Zhou, L.Y., Gao, J.N., Chen, C., Yan, K.W., Ponnusamy, M., Zhang, Y.H., and Li, P.F. (2017). Circular RNA mediates cardiomyocyte death via miRNA-dependent upregulation of MTP18 expression. Cell Death Differ. *24*, 1111–1120.
31. Liu, X., Liu, H., Wang, M., Li, R., Zeng, J., Mo, D., Cong, P., Liu, X., Chen, Y., and He, Z. (2019). Disruption of the ZBED6 binding site in intron 3 of IGF2 by CRISPR/Cas9 leads to enhanced muscle development in Liang Guang Small Spotted pigs. Transgenic Res. *28*, 141–150.
32. Mu, Q., Wang, L., Yu, F., Gao, H., Lei, T., Li, P., Liu, P., Zheng, X., Hu, X., Chen, Y., et al. (2015). Imp2 regulates GBM progression by activating IGF2/PI3K/Akt pathway. Cancer Biol. Ther. *16*, 623–633.
33. Drögemüller, C., Drögemüller, M., Leeb, T., Mascarello, F., Testoni, S., Rossi, M., Gentile, A., Damiani, E., and Sacchetto, R. (2008). Identification of a missense mutation in the bovine ATP2A1 gene in congenital pseudomyotonia of Chianina cattle: an animal model of human Brody disease. Genomics *92*, 474–477.
34. Chacko, E., and Ranganathan, S. (2009). Genome-wide analysis of alternative splicing in cow: implications in bovine as a model for human diseases. BMC Genomics *10* (*Suppl 3*), S11.
35. Trees, A.J., Graham, S.P., Renz, A., Bianco, A.E., and Tanya, V. (2000). Onchocerca ochengi infections in cattle as a model for human onchocerciasis: recent developments. Parasitology *120* (*Suppl*), S133–S142.
36. Visscher, P.M., and Goddard, M.E. (2011). Cattle gain stature. Nat. Genet. *43*, 397–398.
37. Chauveau, C., Rowell, J., and Ferreiro, A. (2014). A rising titan: TTN review and mutation update. Hum. Mutat. *35*, 1046–1059.
38. Kellermayer, D., Smith, J.E., 3rd, and Granzier, H. (2019). Titin mutations and muscle disease. Pflugers Arch. *471*, 673–682.
39. Wilusz, J.E., and Sharp, P.A. (2013). Molecular biology. A circuitous route to noncoding RNA. Science *340*, 440–441.
40. Yang, Y., Fan, X., Mao, M., Song, X., Wu, P., Zhang, Y., Jin, Y., Yang, Y., Chen, L.L., Wang, Y., et al. (2017). Extensive translation of circular RNAs driven by N^6-methyladenosine. Cell Res. *27*, 626–641.
41. Salmena, L., Poliseno, L., Tay, Y., Kats, L., and Pandolfi, P.P. (2011). A ceRNA hypothesis: the Rosetta Stone of a hidden RNA language? Cell *146*, 353–358.

42. Qi, X., Zhang, D.H., Wu, N., Xiao, J.H., Wang, X., and Ma, W. (2015). ceRNA in cancer: possible functions and clinical implications. J. Med. Genet. *52*, 710–718.

43. Tang, Y., Wang, J., Lian, Y., Fan, C., Zhang, P., Wu, Y., Li, X., Xiong, F., Li, X., Li, G., et al. (2017). Linking long non-coding RNAs and SWI/SNF complexes to chromatin remodeling in cancer. Mol. Cancer *16*, 42.

44. Fan, C., Tang, Y., Wang, J., Xiong, F., Guo, C., Wang, Y., Zhang, S., Gong, Z., Wei, F., Yang, L., et al. (2017). Role of long non-coding RNAs in glucose metabolism in cancer. Mol. Cancer *16*, 130.

45. Guo, L.L., Song, C.H., Wang, P., Dai, L.P., Zhang, J.Y., and Wang, K.J. (2015). Competing endogenous RNA networks and gastric cancer. World J. Gastroenterol. *21*, 11680–11687.

46. Ma, M., Wang, X., Chen, X., Cai, R., Chen, F., Dong, W., Yang, G., and Pang, W. (2017). MicroRNA-432 targeting E2F3 and P55PIK inhibits myogenesis through PI3K/AKT/mTOR signaling pathway. RNA Biol. *14*, 347–360.

47. Chen, L., Kong, G., Zhang, C., Dong, H., Yang, C., Song, G., Guo, C., Wang, L., and Yu, H. (2016). MicroRNA-432 functions as a tumor suppressor gene through targeting E2F3 and AXL in lung adenocarcinoma. Oncotarget *7*, 20041–20053.

48. Liu, Y., Lu, C., Zhou, Y., Zhang, Z., and Sun, L. (2018). Circular RNA hsa_circ_0008039 promotes breast cancer cell proliferation and migration by regulating miR-432-5p/E2F3 axis. Biochem. Biophys. Res. Commun. *502*, 358–363.

49. Li, G., Luo, W., Abdalla, B.A., Ouyang, H., Yu, J., Hu, F., Nie, Q., and Zhang, X. (2017). miRNA-223 upregulated by MYOD inhibits myoblast proliferation by repressing IGF2 and facilitates myoblast differentiation by inhibiting ZEB1. Cell Death Dis. *8*, e3094.

50. Wei, C., Wu, M., Wang, C., Liu, R., Zhao, H., Yang, L., Zhang, S., Yuan, Z., Liu, Z., Hu, S., et al. (2018). Long Noncoding RNA Lnc-SEMT Modulates IGF2 Expression by Sponging miR-125b to Promote Sheep Muscle Development and Growth. Cell. Physiol. Biochem. *49*, 447–462.

51. Afshar, S., Najafi, R., Sedighi Pashaki, A., Sharifi, M., Nikzad, S., Gholami, M.H., Khoshghadam, A., Amini, R., Karimi, J., and Saidijam, M. (2018). MiR-185 enhances radiosensitivity of colorectal cancer cells by targeting IGF1R and IGF2. Biomed. Pharmacother. *106*, 763–769.

52. Fei, S., Cao, L., and Pan, L. (2018). microRNA-3941 targets IGF2 to control LPS-induced acute pneumonia in A549 cells. Mol. Med. Rep. *17*, 4019–4026.

53. Liu, J., Jia, Y., Jia, L., Li, T., Yang, L., and Zhang, G. (2019). MicroRNA 615-3p Inhibits the Tumor Growth and Metastasis of NSCLC via Inhibiting IGF2. Oncol. Res. *27*, 269–279.

54. Yi, F., Shang, Y., Li, B., Dai, S., Wu, W., Cheng, L., and Wang, X. (2017). MicroRNA-193-5p modulates angiogenesis through IGF2 in type 2 diabetic cardiomyopathy. Biochem. Biophys. Res. Commun. *491*, 876–882.

55. Van Laere, A.S., Nguyen, M., Braunschweig, M., Nezer, C., Collette, C., Moreau, L., Archibald, A.L., Haley, C.S., Buys, N., Tally, M., et al. (2003). A regulatory mutation in IGF2 causes a major QTL effect on muscle growth in the pig. Nature *425*, 832–836.

56. Erbay, E., Park, I.H., Nuzzi, P.D., Schoenherr, C.J., and Chen, J. (2003). IGF-II transcription in skeletal myogenesis is controlled by mTOR and nutrients. J. Cell Biol. *163*, 931–936.

57. Zhang, Q., Wang, Q., Gong, J., Du, J., Zhang, Y., and Zhao, X. (2018). Yak IGF2 Promotes Fibroblast Proliferation Via Suppression of IGF1R and PI3KCG Expression. Genes (Basel) *9*, 169.

58. Liu, M., and Zhang, S. (2011). Amphioxus IGF-like peptide induces mouse muscle cell development via binding to IGF receptors and activating MAPK and PI3K/Akt signaling pathways. Mol. Cell. Endocrinol. *343*, 45–54.

59. Codina, M., García de la serrana, D., Sánchez-Gurmaches, J., Montserrat, N., Chistyakova, O., Navarro, I., and Gutiérrez, J. (2008). Metabolic and mitogenic effects of IGF-II in rainbow trout (Oncorhynchus mykiss) myocytes in culture and the role of IGF-II in the PI3K/Akt and MAPK signalling pathways. Gen. Comp. Endocrinol. *157*, 116–124.

60. Li, H., Wei, X., Yang, J., Dong, D., Huang, Y., Lan, X., et al. (2017). Developmental transcriptome profiling of bovine muscle tissue reveals an abundant GosB that regulates myoblast proliferation and apoptosis. Oncotarget *8*, 32083–32100.

OMTN, Volume *18*

Supplemental Information

Circular RNA TTN Acts As a miR-432 Sponge to Facilitate Proliferation and Differentiation of Myoblasts via the IGF2/PI3K/AKT Signaling Pathway

Xiaogang Wang, Xiukai Cao, Dong Dong, Xuemei Shen, Jie Cheng, Rui Jiang, Zhaoxin Yang, Shujun Peng, Yongzhen Huang, Xianyong Lan, Ibrahim Elsaeid Elnour, Chuzhao Lei, and Hong Chen

Supplemental Information

Table S1. Primers for qPCR

Name	Forward primer 5’→3’	Reverse primer 5’→3’
CircTTN	AAAGAACTTCCACCTCCTAAA	CGACAACCTTTTTAGCATCTT
Bta-miR-432	CGGCTCTTGGAGTAGGTCATT	GCAGGGTCCGAGGTATTC
miR-432-RT	GTCGTATCCAGTGCAGGGTCCGAGGTATTCGCACTGGATACGACCCACCC	
U6	GCTTCGGCAGCACATATACTAAAAT	CGCTTCACGAATTTGCGTGTCAT
GAPDH	TGAGGACCAGGTTGTCTCCTGCG	CACCACCCTGTTGCTGTAGCCA
MYF5	CTGCAGGAGCTGCTTAGGGA	AGGCATGCCATCAGAGCAAC
MYOD	CGCTCCAACTGTTCCGACG	AAAGGGCATGTGGGAAGGAG
MYOG	AGGGGATCATCTGCTCCCAG	ATCCCGGCAGACAATCTCAG
MYHC	GGAGGCTGATGAACAAGCCAA	GAAGTCTCGGGTCTTTGCTCT
PCNA	TCCAGAACAAGAGTATAGC	TACAACAGCATCTCCAAT
CDK2	TCTTTGCTGAGATGGTGACCC	CATCTTCATCCAGGGGAGGC
CyclinD1	CCGTCCATGCGGAAGATC	CAGGAAGCGGTCCAGGTAG
TTN	AGGGCAATTACAGATTGGTGTG	AAACTGGCAGGTCATGGTACA
IGF2	CCGCAGGAGCTGAGACATCAA	CGGTCCCCACAGACAAACTG
IRS1	TTCGCCTACCATTTCCCACC	TGGGTAAGCGGGCATCATTT
PI3K	AGCGCTGAGCAGTGTATCTT	AACCACGGGGCTCTTGAAAT
PDK1	GAAAATGCTAGGCGTCTGTGT	CACCTCCTCGGTCACTCATC
AKT	TCCCCCAGTTCTCCTACTCG	TCCTCTCCATCCTGTGTTGG

Table S2. Primers for vector construction

Name	Primer sequence 5’→3’
PCD2.1-circTTN-F	GGGGTACCAAGAGACCCAGGTAGAAGCTA
PCD2.1-circTTN-R	CGGGATCCCGTAATCTTCCTCTGGTTCCT
psiCHECK2-circTTN-F	CCGCTCGAGAAGAGACCCAGGTAGAAGCTA
psiCHECK2-circTTN-R	ATAAGAATGCGGCCGCCGTAATCTTCCTCTGGTTCCT
psiCHECK2-miR-432-Sensor-F	TCGACCACCCAATGACCTACTCCAAGACCACCCAATGA CCTACTCCAAGA
psiCHECK2-miR-432-Sensor-R	GGCCTCTTGGAGTAGGTCATTGGGTGGTCTTGGAGTAG GTCATTGGGTGG
psiCHECK2-IGF2-W-F	CCGCTCGAGTGCAGGTAGGCTTGTCCTTG
psiCHECK2-IGF2-W-R	ATAAGAATGCGGCCGCTTGGAGTGTGGGGGTGTTTT
IGF2-MUT-F	CTGCTCCTCCCAAGAGTTTCCATCA
IGF2-MUT-R	TGATGGAAACTCTTGGGAGGAGCAG
psiCHECK2-IGF1-W-F	GAAAGAGTCTGGCCAAAACGG
psiCHECK2-IGF1-W-R	CAGATGTTTGTTTCTTCAGCGAG
IGF1-MUT-F	GCTCCCGCATTATGCCTTTAGGGAA
IGF1-MUT-R	TTCCCTAAAGGCATAATGCGGGAGC

Text S1. Sequence of cattle circTTN

>circTTN

TGACCAAAAAAGCTGTGAAGAAAGATGCTAAAAAGGTTGTCGCAAAGCCCAAGGAAGAG

GCACCGCCACCTAAAGTTATAGAGGTTCCCAAGAAGCCACCACGTCCTACTGCCTTAATTCC

AGCAGAAGCACCTGAAATTATTGATGTATCCTCCAAGGCTGAAGAAGTGAAAATAACGACC

ATAACCAGAAAGAAAGAGGTTCAGAAAGAAAAAGAAGCTGTGTATGAGAGAAAGCAAGC

AGTCTACGAGGAGAAGAAAGTTTACATAGAATCTTTGGAAGAACCTTACGATGAGCTAGAG

GTGGAAACGTATACGGAGCCATATGAAGAACCTTATTATGAGGAACCAGAGGAAGATTACG

AAGAGACCCAGGTAGAAGCTAAGAGGGAAGTTCATGAGGAATGGGAAGAAGATTTTGAAG

AAGGGCAAGAATACTATGAAAGGGAGGAAGGTTATGACGAAGGAGAGGAAGAGTGGGAG

GAGACTTACCAAGAGAGAGAAGTCGTCCAAGTTCAAAAGGAGGTTTATGAAGAATCACGT

GAGAGGAAAATTCCAGCAAAAGTACCTGAAAAGAAAGAACTTCCACCTCCTAAAGTTGTA

AAGAAGCCTGTAGTTGAAAAAATTGAAAAGACTTCTCGAAGAATGGAGGAAGAAAAAGTT

CAAGTCACCAAAG

Figure S1

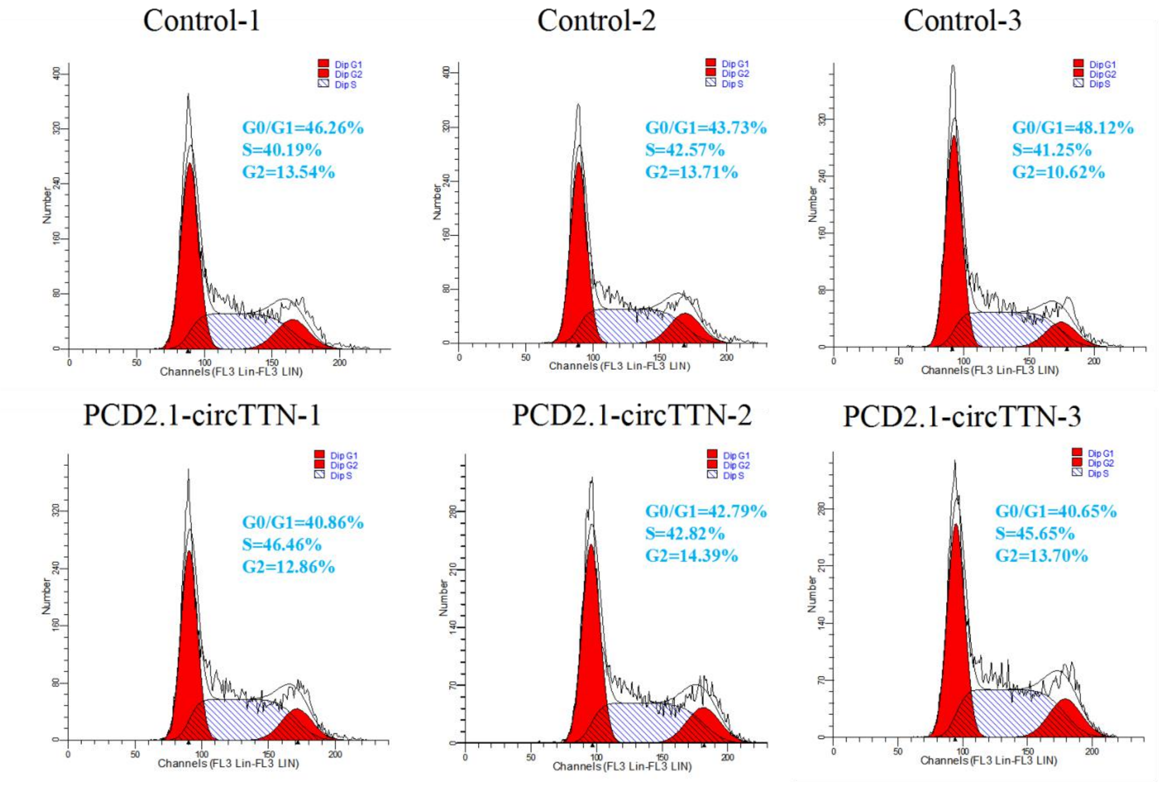

Figure S1. Bovine primary myoblasts were transfected with PCD2.1-circTTN, and cell phases were analyzed by flow cytometry

Figure S2

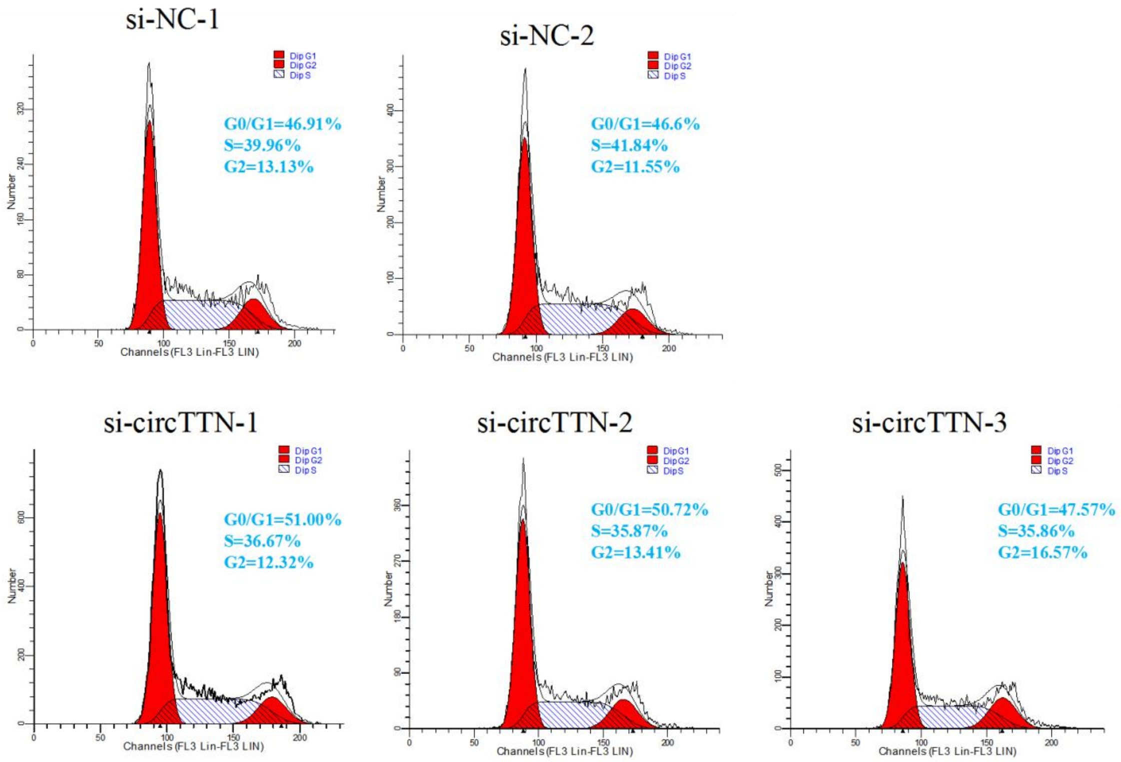

Figure S2. Bovine primary myoblasts were transfected with si-circTTN, and cell phases were analyzed by flow cytometry

Figure S3

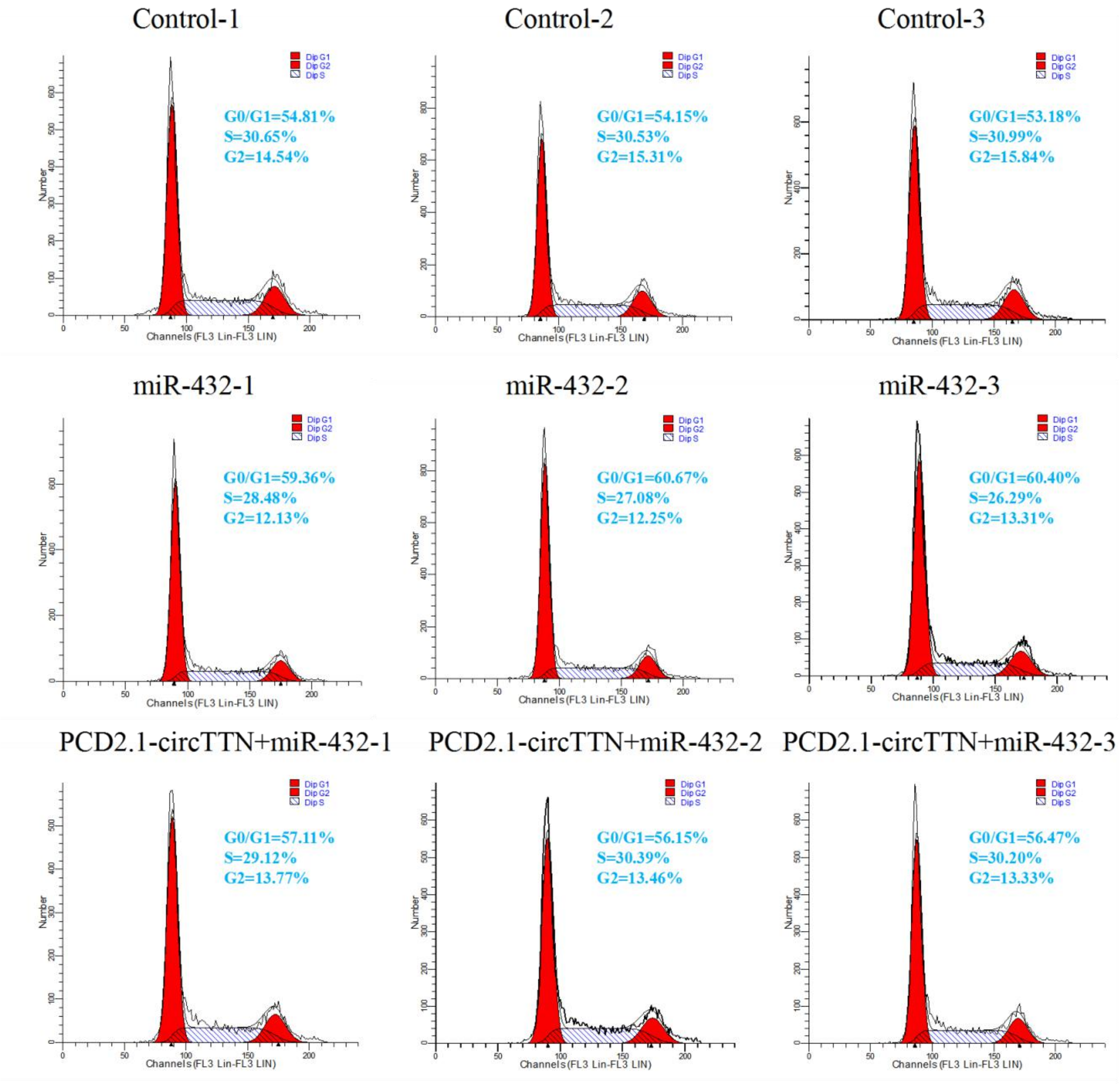

Figure S3. Bovine primary myoblasts were transfected with miR-432 mimic and/or PCD2.1-circTTN, and cell phases were analyzed by flow cytometry

Figure S4

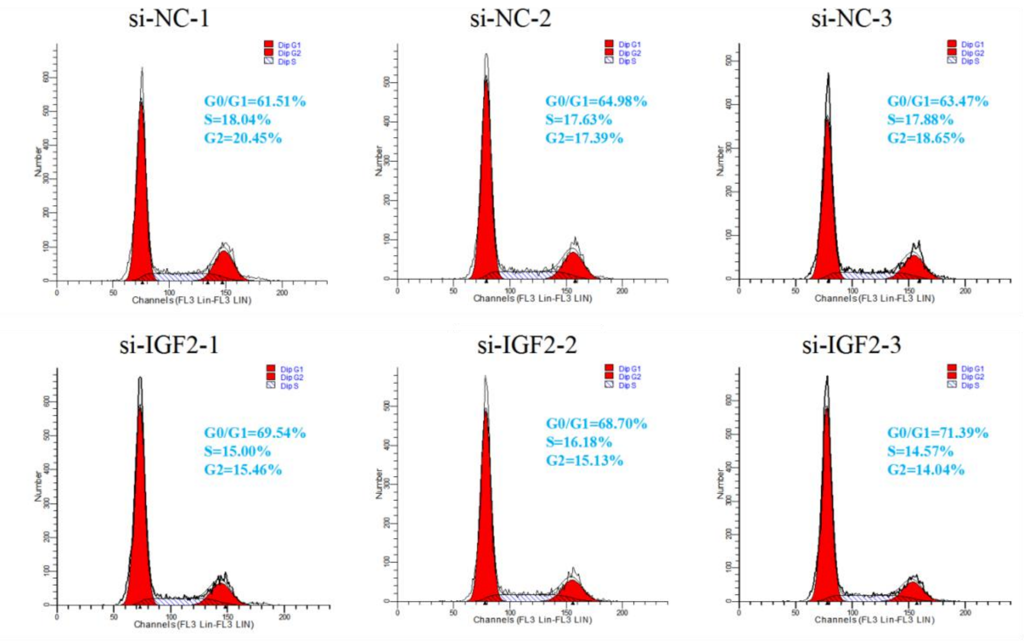

Figure S4. Bovine primary myoblasts were transfected with si-IGF2, and cell phases were analyzed by flow cytometry

Figure S5

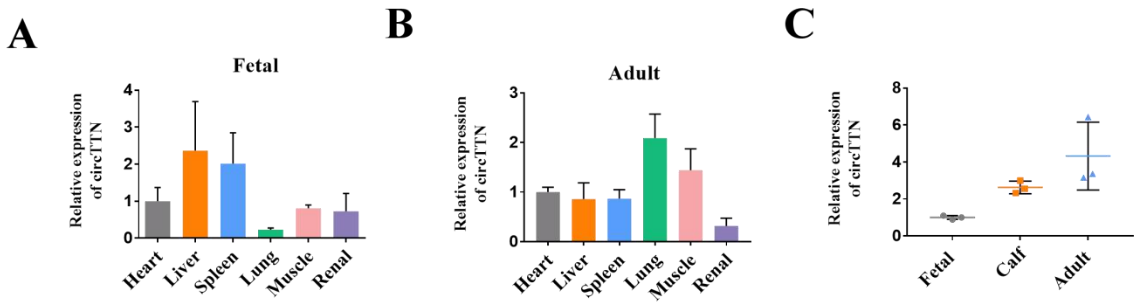

(A and B) The expression of circTTN in different tissues of Qinchuan cattle at embryonic (A) and adult (B) stage. (C) The expression of circTTN in skeletal muscle of cattle at the fetal stage, calf stage and adult stage.

Figure S6

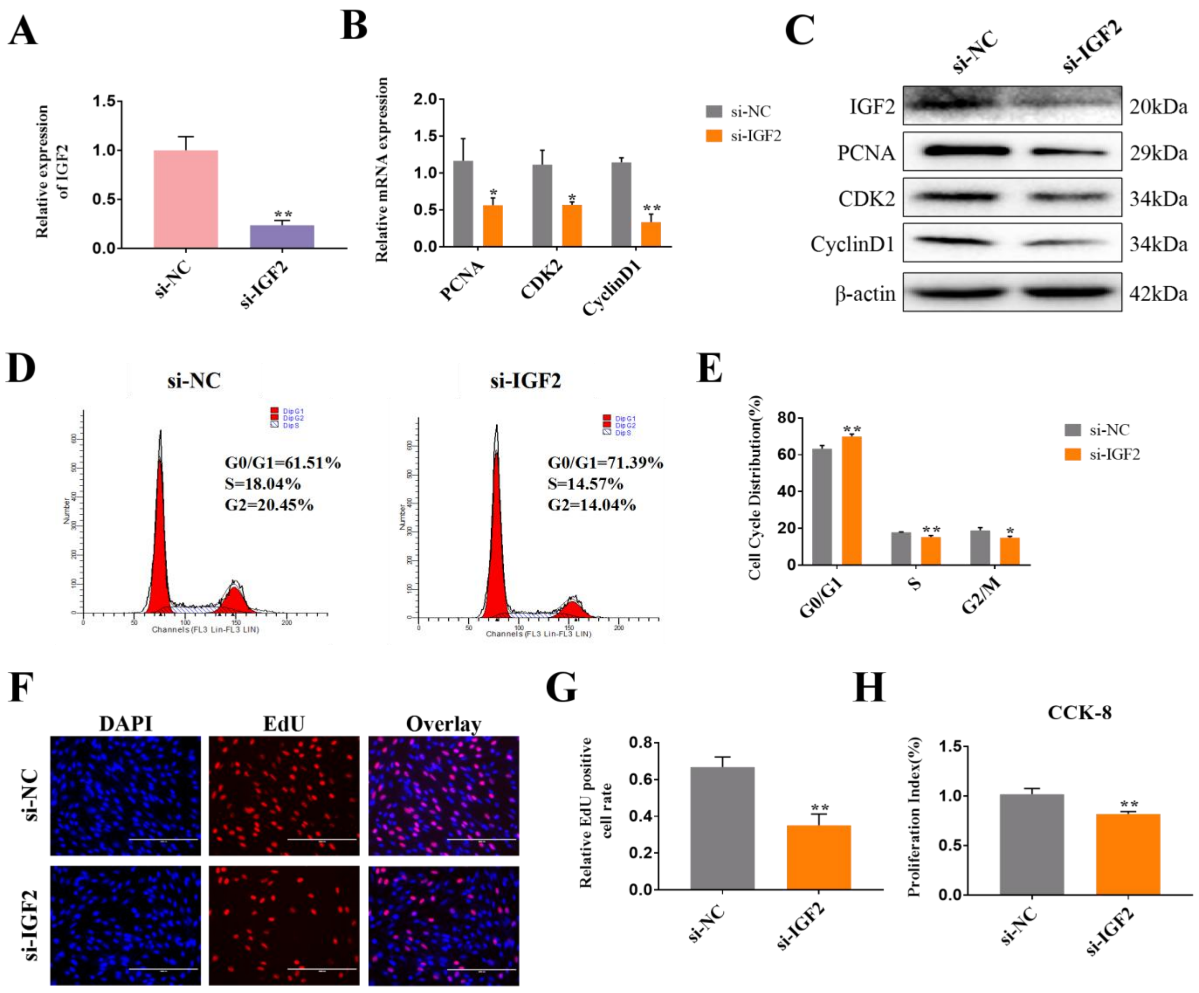

Figure S6 Effect of *IGF2* Knockdown on Proliferation of Bovine Primary Myoblasts

(A) The interference efficiency of the si-*IGF2* is detected by RT-qPCR. (B) Detection of the expression levels of the cell proliferation genes mRNA (*PCNA*, *CDK2*, *Cyclin D1*) by RT-qPCR. (C) Detection of PCNA, CDK2, and Cyclin D1 protein expression levels by western blot analysis, β-actin acts as an internal control gene. (D and E) Bovine primary myoblasts were transfected with si-*IGF2*, and cell phases were analyzed by flow cytometry (D) and counted (E). (F and G) Cell proliferation was detected by 5-ethynyl-2'-deoxyuridine (EdU) (F) and counted using Image J (G). Scale bar indicates 200 μm. (H) Cell proliferation index was detected by cell counting kit-8 (CCK-8) assay. Data are presented as means ± SEM for three individuals. *$P < 0.05$; **$P < 0.01$.

Figure S7

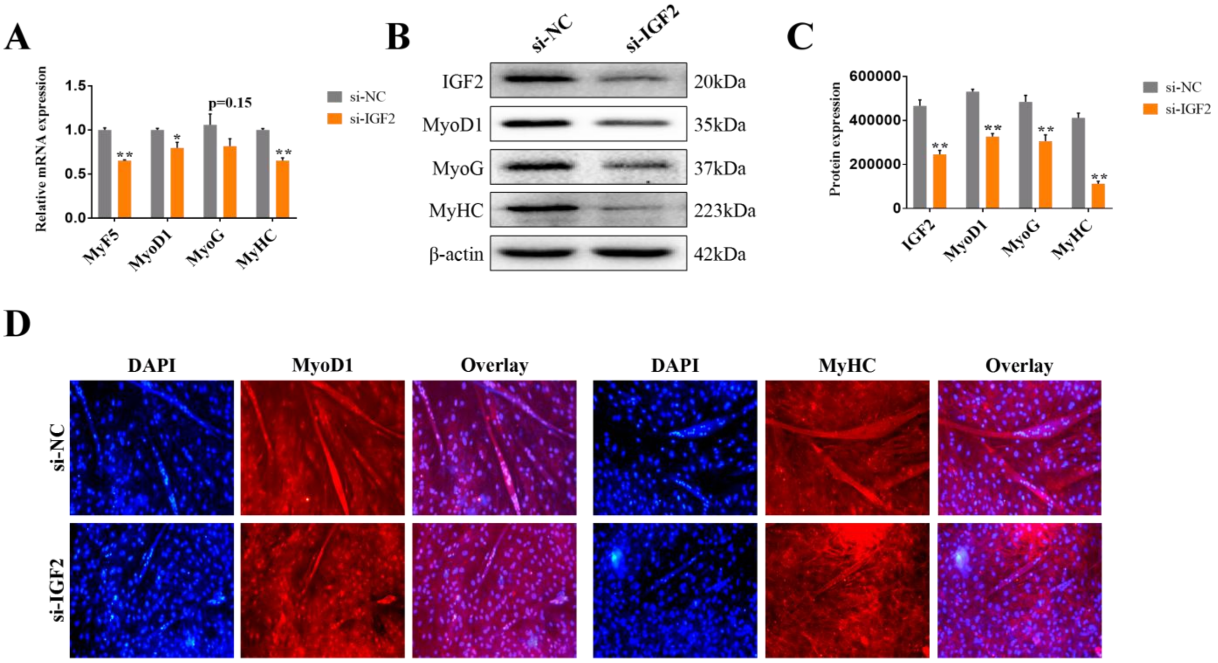

Figure S7 Effect of *IGF2* Knockdown on Differentiation of Bovine Primary Myoblasts

(A) Transfection of the si-*IGF2* and the expression levels of myogenic differentiation marker genes *MyF5*, *MyoD1*, *MyoG*, and *MyHC* mRNA were detected by RT-qPCR. (B and C) MyoD1, MyoG, and MyHC protein levels were detected by western blot analysis (B), and protein band density was also analyzed by ImageJ (C). (D) Bovine primary myoblasts were transfected with si-*IGF2* and cell differentiation was measured by immunofluorescence (MyoD1 (left) and MyHC (right)) and observed under a fluorescence microscope. Scale bar indicates 200 µm. Data are presented as means ± SEM for three individuals. *$P < 0.05$; **$P < 0.01$.
